# Cloxiquine, a traditional antituberculosis agent, suppresses the growth and metastasis of melanoma cells through activation of PPARγ

**DOI:** 10.1038/s41419-019-1644-8

**Published:** 2019-05-28

**Authors:** Wenxiang Zhang, Wei Shao, Zhewen Dong, Shiyao Zhang, Chang Liu, Siyu Chen

**Affiliations:** 1 State Key Laboratory of Natural Medicines, China Pharmaceutical University, Nanjing, Jiangsu China; 2 School of Life Science and Technology, China Pharmaceutical University, Nanjing, Jiangsu China; 3 State key Laboratory of Pharmaceutical Biotechnology, Nanjing University, Nanjing, Jiangsu China

**Keywords:** Cancer metabolism, Phenotypic screening, Melanoma

## Abstract

Melanoma is one of the most aggressive skin cancers and 5-year survival rate is only 4.6% for metastatic melanoma patients. Current therapies, especially those involving clinical chemotherapy drugs, have achieved remarkable advances. However, their side effects, such as bone marrow suppression, limit the effectiveness of available pharmacological therapies. Therefore, exploring new antimelanoma drugs with less toxicity is critical for the treatment of melanoma. In the present study, we aimed to identify the antimelanoma drugs with ability to repress the proliferation of melanoma cells by using a high-content screening of FDA-approved drug libraries. We found that cloxiquine (CLQ), a traditional antituberculosic drug, exhibited strong inhibitory effects on the growth and metastasis of melanoma cells both in vivo and in vitro. In contrast, CLQ at the tested doses did not show any apparent toxicity in normal melanocytes and in the liver. At the metabolic level, treatment with CLQ decreased glycolysis, thus potentially inhibiting the “Warburg effect” in B16F10 cells. More importantly, combination of CLQ and 2-deoxyglucose (2-DG), a well-known glycolysis inhibitor, did not show a synergistic effect on the tumor growth and metastasis, indicating that inhibition of glycolysis is potentially involved in mediating CLQ’s antimelanoma function. Bioinformatics analyses revealed that peroxisome proliferator-activated receptor-gamma (PPARγ) served as a potential CLQ target. Mechanistically, CLQ stimulated the transcription and nuclear contents of PPARγ. Furthermore, the specific PPARγ inhibitor GW9662 or PPARγ shRNA partially abolished the effects of CLQ. Collectively, our findings demonstrate that CLQ has a great potential in the treatment of melanoma through activation of PPARγ.

## Introduction

Melanoma is one of the most aggressive skin cancers, and it originates from the malignant transformation of melanocytes^1,2^. Risk factors for the occurrence of melanoma include genetic susceptibility, sex, age, skin pigmentation, tanning ability, nevus count, freckling, and psychological health^3,4^. In addition, environmental features, such as occupation, latitude, and the level of ozone layer, have been identified to influence the morbidity of melanoma^4^. In 2017, estimated 87,110 new melanoma cases were diagnosed in the USA. This disease caused nearly 13,950 deaths, which accounts for 90% of all skin cancer-induced deaths^5^. Moreover, the incidence of melanoma has increased by 3.1% per year and has doubled each decade, representing a great threat to public health and entire social economies^6^.

The proliferation and metastasis of malignant cells are the major underlying biological processes of melanoma cancer. Although clinical chemotherapy drugs, including cisplatin and nitrogen mustard, antagonize melanoma progression, their toxic side effects, such as bone marrow and immune suppression, limit the effectiveness of currently available pharmacological therapies^7–9^. On the other hand, with advances in the genotype-directed treatment, specific and highly effective small-molecule inhibitors targeting BRAF and MEK mutants have been developed and used in the treatment of BRAF-mutant metastatic melanoma patients. However, drug resistance occurs and renders these drugs ineffective^10^. Similarly, new immunotherapeutic strategies, such as using antibodies against cytotoxic T-lymphocyte-associated antigen 4 (CTLA4, ipilimumab) and programmed cell death 1 (PD-1) or programmed death ligand 1 (PD-L1, pembrolizumab or nivolumab), are beneficial to only a subset of the patients in clinics^11,12^. Therefore, a new strategy aiming to suppress melanoma progression may have a high value in the therapy of melanoma cancer.

Given that the de novo development of new drugs is a time-consuming and costly process, drug reposition serves as an effective and innovative approach to hasten the development cycles, especially those in cancer therapeutics. More importantly, repositioned drugs have the minimal risk in the clinical uses due to the comprehensive pharmacological, safety and toxicological data^13^. For instance, as an anti-diabetic drug, metformin decreases human cancer incidence, thus improving the survival of cancer patients^14^. Therefore, we focused on drug reposition and presented a CCK-8 assay-based drug screening in mouse B16F10 melanoma cells using the FDA-approved drug library (composed of 1430 small compounds). Among these, we found that cloxiquine (CLQ) exhibited strong antimelanoma properties. CLQ is a well-known anti-infection and antibacterial drug clinically used in the treatment of tuberculosis. Notably, CLQ has a unique p53-modulating activity that shifts its transactivation from pro-apoptotic to protective responses, including enhancing p21 induction, thereby protecting mice from γ-irradiation-induced gastrointestinal death^15^. In addition, CLQ protects from cardiac ischemia-reperfusion (IR) injury in mice by uncoupling mitochondria and inducing autophagy^16^. However, the beneficial action of CLQ on melanoma and the direct molecular target through which CLQ exerts its antitumor function remain unknown. In our present study, we demonstrated that CLQ exerted strong antimelanoma properties in vitro and in vivo, and the antitumor effects of CLQ were partially, if not totally, mediated by peroxisome proliferator-activated receptor-gamma (PPARγ). Collectively, our findings offer new perspectives for the potential of CLQ in melanoma treatment.

## Results

### Compound screenings identify CLQ as a potential antimelanoma drug

**Fig. 1:**
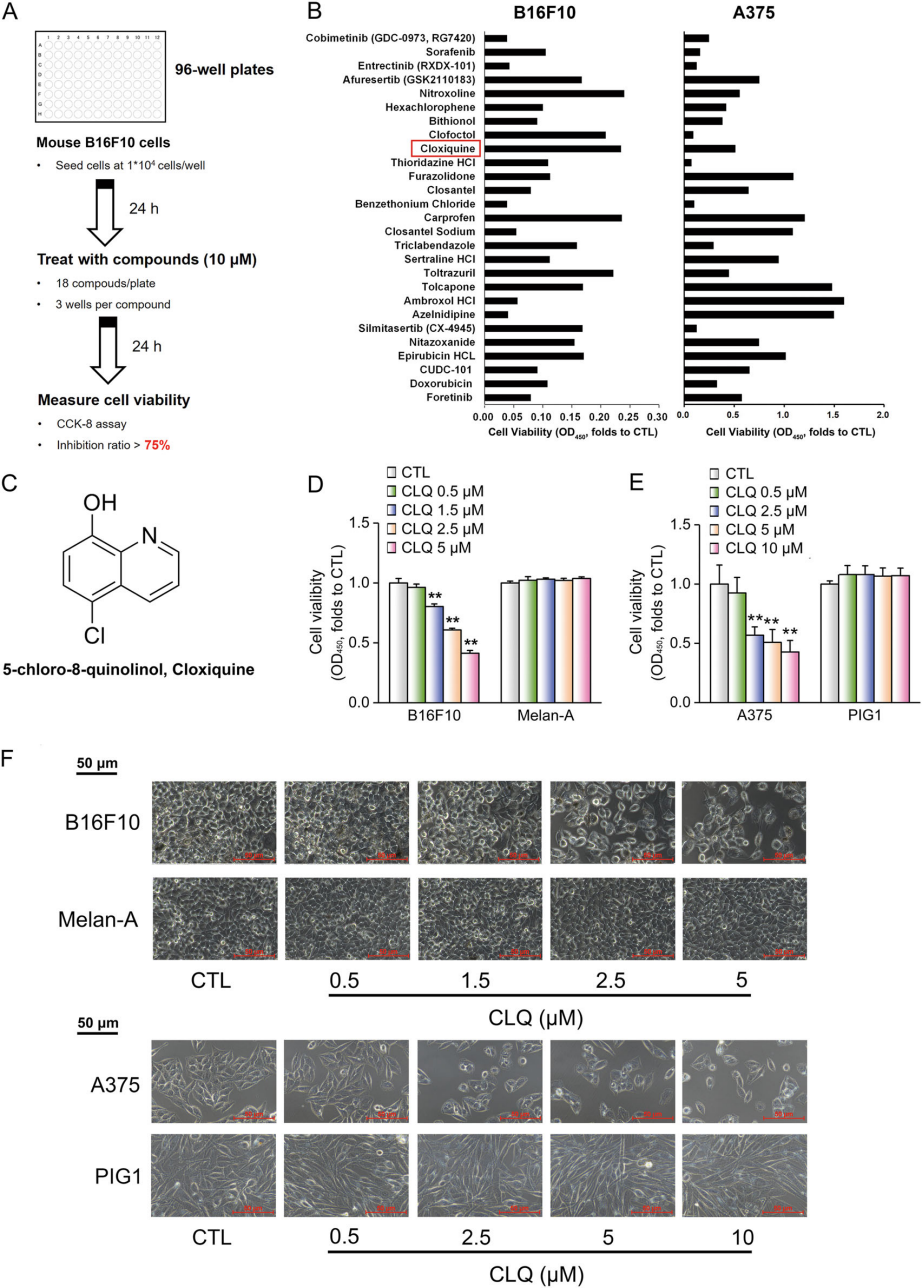
Compound screenings in B16F10 cells identify CLQ as a potential antimelanoma drug. **a** The schematic workflow of the compound screen. **b** Cell viability of melanoma (B16F10 and A375) cells treated with indicated functional drugs. **c** Chemical structure of CLQ. **d**, **e** Mouse B16F10 and Melan-A melanocyte cells, as well as the human A375 and PIG1 cells were treated with the indicated concentrations of CLQ for 24 h. The inhibitory effect of CLQ on cell viability was detected by a CCK-8 assay. **f** Morphological analysis. All values are presented as the mean ± SD from at least three separate experiments. **P < 0.01 vs. CTL

To identify novel inhibitors of melanoma cell growth, 1430 small compounds (10 μM final concentration) from the FDA-approved drug library were screened for their ability to repress cell proliferation of mouse B16F10 cells (Fig. 1a). CCK-8 analysis indicated that 27 drugs exhibited dramatic inhibitory effects on the viability of these cells (inhibition ratio > 75%). For the future translational medicine purpose, we double checked the actions of these drugs on human A375 melanoma cells and found that 19 of which demonstrated similar functions (Fig. 1b). Among them, 18 drugs are already known as antitumor agents or their targets are involved in tumorigenesis (Supplementary Table 1), and only CLQ (Fig. 1c), a classic antituberculosis drug, has never been reported to be functional for the cancer therapy. Therefore, it is interesting to explore potential antimelanoma effects of CLQ in the present study. Importantly, although CLQ suppressed both B16F10 and A375 cell growth in a dose-dependent manner, it did not affect the viability of normal Melan-A and PIG1 melanocytes, suggesting that it is safe for non-tumor cells (Fig. 1d, e). These results were confirmed by morphological observations (Fig. 1f).

### CLQ inhibits the proliferation of melanoma cells and suppresses tumor growth in a mouse B16F10 melanoma xenograft model

**Fig. 2:**
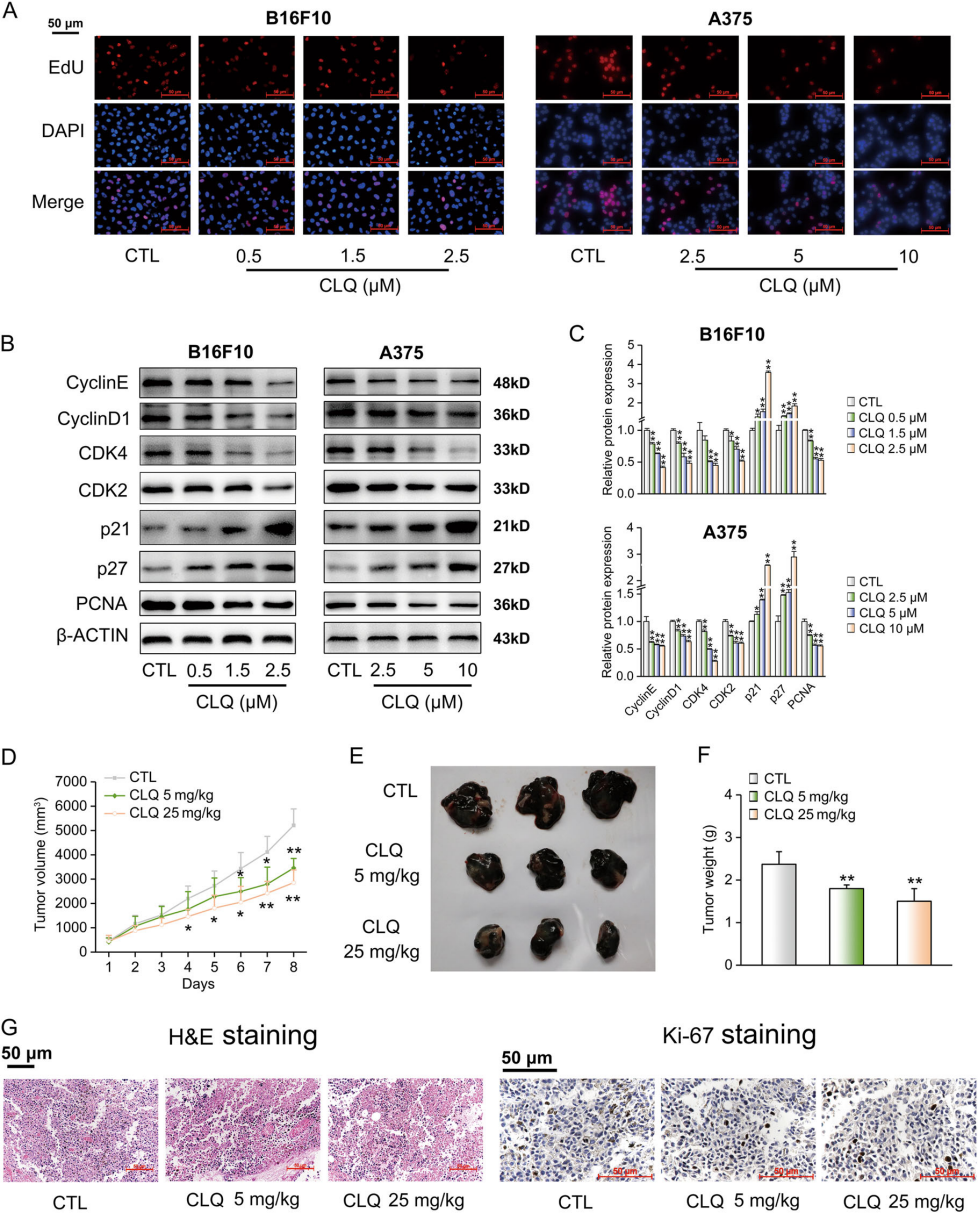
CLQ inhibits the proliferation of melanoma cells and suppresses tumor growth in vivo. B16F10 and A375 cells were treated with the indicated concentrations of CLQ for 24 h. **a** EdU incorporation assay used to measure cell proliferation. **b** Protein expression levels of key regulators involved in cell cycle progression. **c** Quantification for the immunoblots from **b**. All values are presented as the mean ± SD from at least three separate experiments. *P < 0.05 and **P < 0.01 vs. CTL. B16F10 cells (1 × 10^6^ cells per mouse) were injected subcutaneously into the right flank of nude mice. When tumors became palpable on day 7, mice were randomly divided into three groups and were administered either vehicle (olive, *i.p*., daily) or CLQ (5 mg/kg, 25 mg/kg, *i.p*., daily) for 8 days. *n* = 5 for each group. **d** Subcutaneous tumor volumes were measured at the indicated days. **e** Representative tumor images. **f** Tumor weights. **g** H&E staining and immunohistochemistry analysis of Ki-67 from tumor sections. All values are presented as the mean ± SD. *P < 0.05 and **P < 0.01 vs. CTL

To further determine the antiproliferative actions of CLQ on melanoma cells, we performed an EdU incorporation assay. We found a significant dose-dependent reduction in cell proliferation after 24-hour (h) CLQ treatment. CLQ at the dose of 2.5 μM or 10 μM exhibited the strongest inhibitory effects on the B16F10 cells (inhibition by 73%) or the A375 cells (inhibition by 64%), respectively (Fig. 2a and Supplementary Fig. 1). At the molecular level, CLQ reduced the protein expression of PCNA, a hallmark of cell proliferation (Fig. 2b). As is known, activated cell proliferation is tightly associated with accelerated cell cycle progression, which is interlocked by the coordination between positive regulators CDK/Cyclin complex and negative regulators of the CDKN1 family (e.g., p27 and p21)^17^. Coincided with this principle, we found that CLQ decreased the protein expression levels of Cyclin E, Cyclin D1, CDK4 and CDK2 in a concentration-dependent manner. In contrast, the protein levels of p27 and p21 were correspondingly increased by CLQ treatment (Fig. 2b, c). Next, we used B16F10 tumor xenograft models to evaluate the suppressive effect of CLQ in vivo. As shown in Fig. 2d, the average tumor volume in the control group reached 5212.63 mm^3^, whereas 5 mg/kg and 25 mg/kg CLQ treatments remarkably decreased tumor volume by 66.37 and 54.79%, respectively, when compared to the control group. Consistently, the xenograft tumor weights were reduced by 75.91 and 63.41% (Fig. 2e, f). Histologically, hematoxylin and eosin (H&E) staining analysis showed that the tumors were composed of densely packed cells from the control group, whereas the administration of CLQ significantly decreased the tumor cell density and blurred tumor cell borders. Immunohistochemistry (IHC) analysis indicated that the number of Ki-67-positive cells gradually reduced in response to CLQ administration (Fig. 2g). In contrast, we did not observe any hepatotoxicity in CLQ-treated mice, as evidenced by modest alterations in serum alanine transaminase (ALT) and aspartate transaminase (AST) levels (Supplementary Fig. 2a). Coincided with these results, we found that the morphology of liver cells was clear and the intracellular margin was discernable, as revealed by H&E staining. At the meanwhile, terminal-deoxynucleoitidyl transferase mediated nick end labeling (TUNEL) assays showed barely DNA fragmentation in the liver sections of these mice (Supplementary Fig. 2b, c). Both these staining results suggest that CLQ is quite safe when administered in vivo.

### CLQ inhibits the migration of melanoma cells and suppresses tumor metastasis in mouse B16F10 melanoma lung metastatic model

**Fig. 3:**
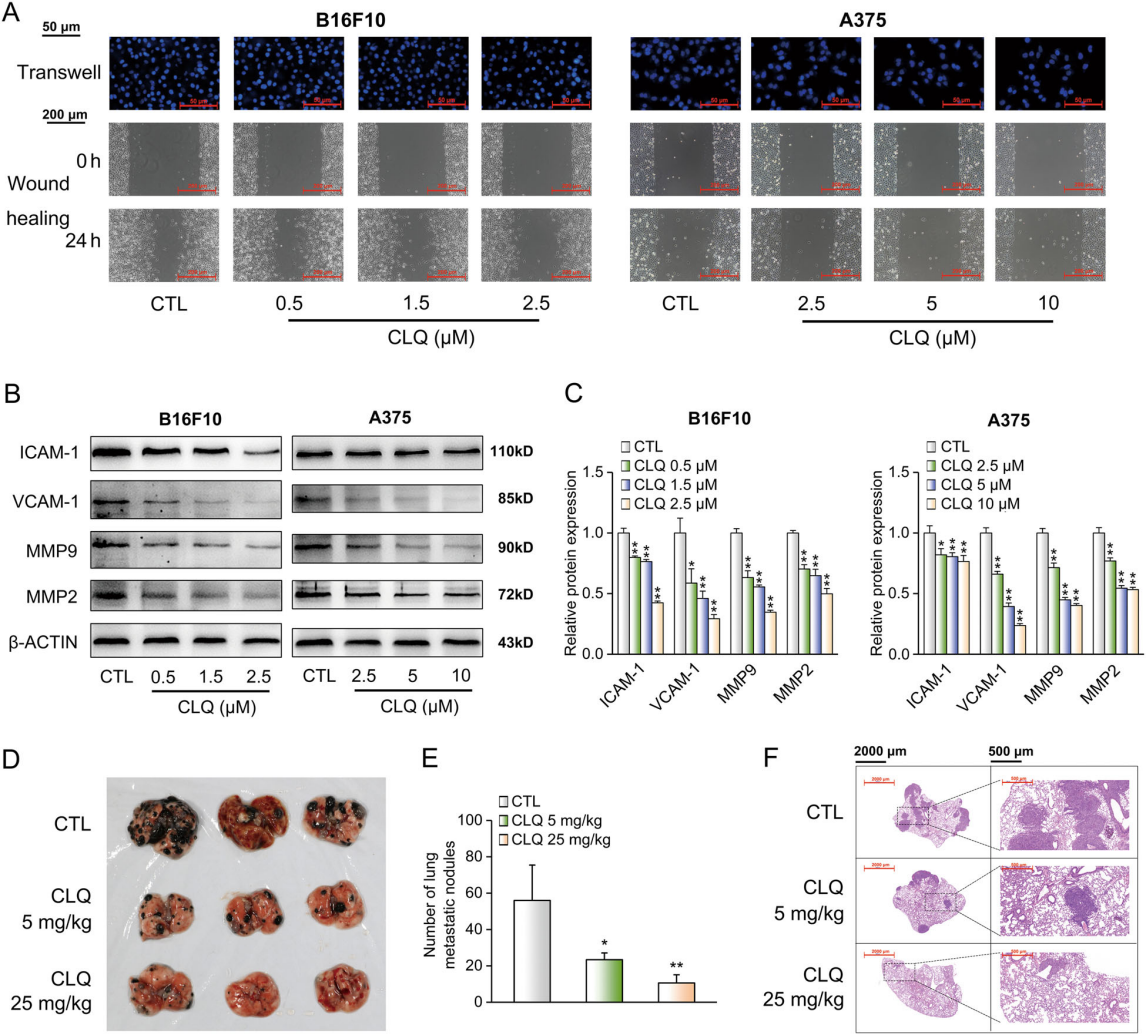
CLQ inhibits the migration of melanoma cells and suppresses tumor metastasis in vivo. B16F10 and A375 cells were treated with the indicated concentrations of CLQ for 24 h. **a** Determination of VSMC migration by transwell chamber (top) and wound-healing (bottom) assays. **b** Protein expression levels of ICAM-1, VCAM-1 and MMPs. **c** Quantification for the immunoblots from **b**. All values are presented as the mean ± SD from at least three separate experiments. *P < 0.05 and **P < 0.01 vs. CTL. B16F10 cells (1.5 × 10^5^ cells per mouse) were injected intravenously. Seven days later, mice were randomly divided into three groups and were administered either vehicle (olive, i.p., daily) or CLQ (5 mg/kg, 25 mg/kg, *i.p*., daily) for 14 days; n = 5 for each group. **d** Macroscopic images, **e** Statistical analyses and **f** H&E staining of lung metastatic nodules. All values are presented as the mean ± SD. *P < 0.05 and **P < 0.01 vs. CTL

Similar to the proliferation results, CLQ decreased the migration rates of B16F10 and A375 cells when assessed by transwell chamber and wound-healing assays (Fig. 3a). The quantitative results were presented in Supplementary Fig. 3. At the molecular level, the expression levels of migration-associated proteins, including ICAM-1, VCAM-1, MMP-9 and MMP-2, were blocked by treatment with CLQ in a dose-dependent manner (Fig. 3b, c). Consistently, by using a B16F10 melanoma metastasis mouse model, we found the number of macroscopic black metastases on the surfaces of the lungs was decreased in the CLQ-treated group when compared with the control group (Fig. 3d, e). H&E staining analysis in lungs showed that the gross morphology of B16F10 melanoma metastasis was alleviated by the administration of CLQ for 14 days (Fig. 3f).

### CLQ suppresses glycolysis in melanoma cells

**Fig. 4:**
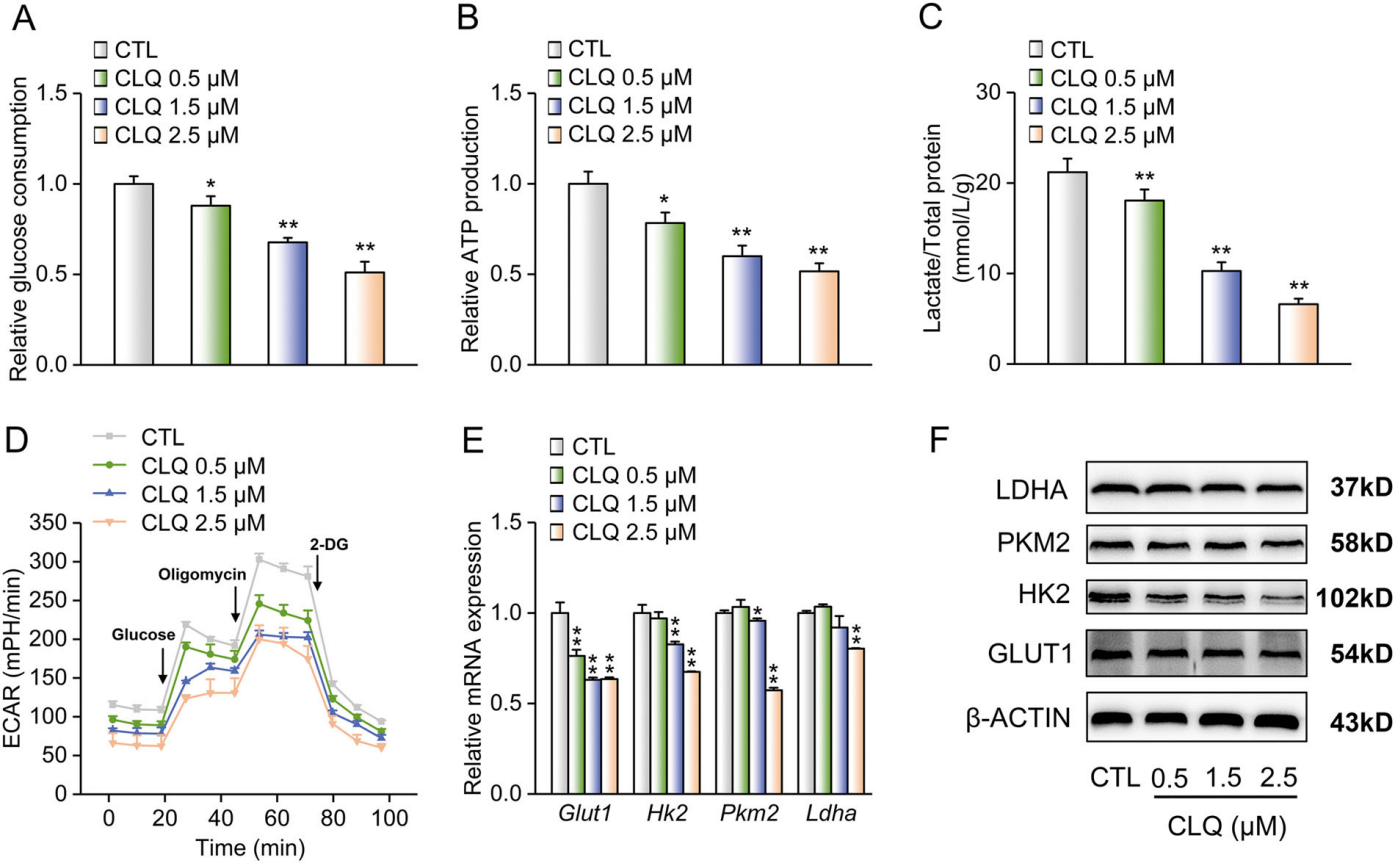
CLQ suppresses the glycolysis in melanoma cells. Mouse B16F10 cells were treated with the indicated concentrations of CLQ for 24 h. **a** Glucose consumption. **b** Relative ATP production. **c** Lactate production. **d** ECAR. **e** RT-qPCR and **f** Western blot analyses of GLUT1, HK2, PKM2 and LDHA expression levels. All values are presented as the mean ± SD from at least three separate experiments. *P < 0.05 and **P < 0.01 vs. CTL

By undergoing aerobic glycolysis (also known as Warburg effect), cancer cells consume glucose to produce lactate, which is the final product of glycolysis, thus providing them with a growth advantage^18,19^. We then investigated if CLQ-inhibited melanoma cell proliferation and migration by blocking glycolysis. As shown in Fig. 4a–c, CLQ increased glucose levels in the culture medium and decreased lactate production, as well as ATP generation, in B16F10 cells. Furthermore, CLQ-treated B16F10 cells exhibited decreased extracellular acidification rate (ECAR), which is an indicator of an overall glycolytic flux (Fig. 4d and Supplementary Fig. 4a). Accordingly, we found a remarkable reduction in the mRNA and protein expression levels of glucose transporter 1 (GLUT1), hexokinase-2 (HK2), pyruvate kinase M2 (PKM2) and lactate dehydrogenase (LDHA), in B16F10 cells after 24-h exposure to CLQ at the indicated doses (Fig. 4e, f and Supplementary Fig. 4b).

### Combination of 2-DG and CLQ does not exhibit synergistic antimelanoma effects

**Fig. 5:**
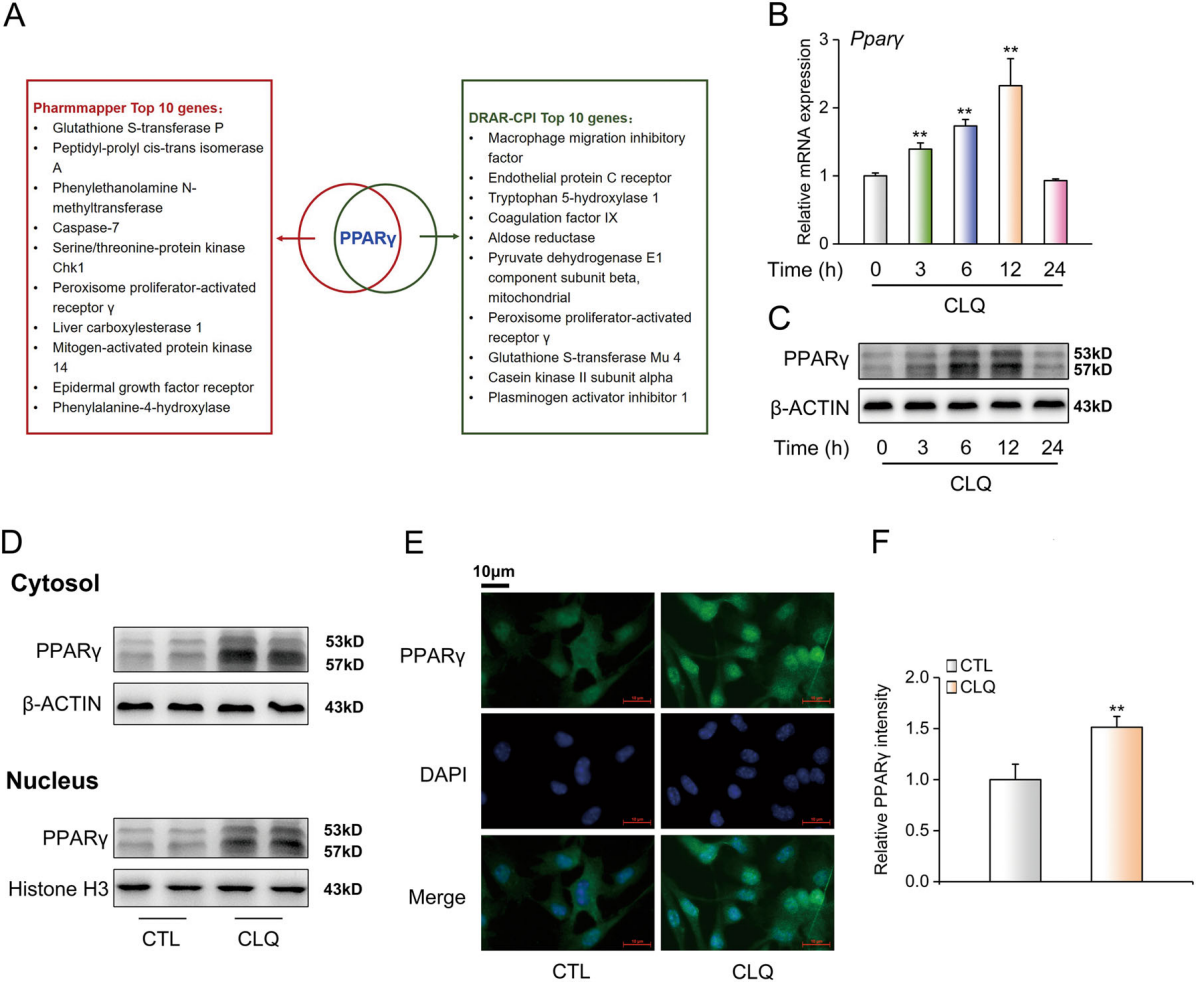
Bioinformatic analyses identify PPARγ as a potential drug target of CLQ. **a** A cluster of the top 10 candidates from bioinformatics analyses using PharmMapper and DRAR-CPI software. To investigate the effect of CLQ on PPARγ expression, B16F10 cells were treated with 1.5 μM CLQ for the indicated time-points. **b** RT-qPCR and (**c**) Western blot analyses of total PPARγ expression levels. **P < 0.01 vs. 0 h. B16F10 cells were treated with 1.5 μM CLQ for 12 h. **d** Protein levels of cytosolic and nuclear PPARγ expression. **e** Immunofluorescence analysis of PPARγ protein expression in B16F10 cells. **f** Quantitative data of (**e**). All values are presented as the mean ± SD from at least three separate experiments. **P < 0.01 vs. CTL

To dissect the role of glycolysis in CLQ-induced impairment of B16F10 growth and metastasis, we treated the cells with either CLQ, 2-deoxyglucose (2-DG, which is a glucose analog and blocks the first step of glycolysis), or their combination. As shown in Supplementary Fig. 5a, while 2-DG and CLQ alone repressed the proliferation and migration of B16F10 cells, the combination of these two reagents did not lead to a further inhibition. The quantitative results were presented in Fig. 5b. Consistently, the protein expression levels associated with accelerated proliferation and migration were reduced, whereas the negative cell cycle regulators p27 and p21 were induced by 2-DG and CLQ (Supplementary Fig. 5c, d). Consistent with in vitro observations, combination of 2-DG and CLQ treatments did not exhibit a synergistic effect on the suppression of tumor growth and metastasis in vivo, compared with 2-DG or CLQ treatment alone (Supplementary Fig. 5e–l).

### Bioinformatics analyses identifies PPARγ as a potential drug target of CLQ

While it has been reported that CLQ has a unique p53-modulating activity and it increases p53 protein expression in human T-cell leukemia MOLT-4 cells, p53 protein levels were modestly regulated upon CLQ treatment in our study (Supplementary Fig. 6a), suggesting that other molecular targets mediate antimelanoma effects of CLQ. To address this concern, we performed bioinformatic analyses by using PharmMapper and DRAR-CPI software. A cluster of the top 10 candidates revealed that PPARγ was a potential CLQ target protein (Fig. 5a and Supplementary Tables 2 and 3). To confirm this result, RT-qPCR and western blot analyses were performed to detect the regulation of CLQ in PPARγ actions in B16F10 cells. Time-course analysis indicated that both PPARγ mRNA and protein expression levels were increased by CLQ in a time-dependent manner up to 12-h treatment and then gradually decreased to the basal level at 24 h (Fig. 5b, c and Supplementary Fig. 6b). Notably, increase of PPARγ nuclear contents represents as a hallmark of PPARγ activation in addition to its expression. Thus, we performed immunoblot analyses and immunofluorescence (ICC) in cellular fractions of B16F10 cells. We found that treatment with CLQ led to an increase of PPARγ both in the cytosol and in nucleus (Fig. 5d–f and Supplementary Fig. 6c), implicating the activation of PPARγ induced by CLQ.

### PPARγ mediates the antimelanoma effects of CLQ in B16F10 cells

**Fig. 6:**
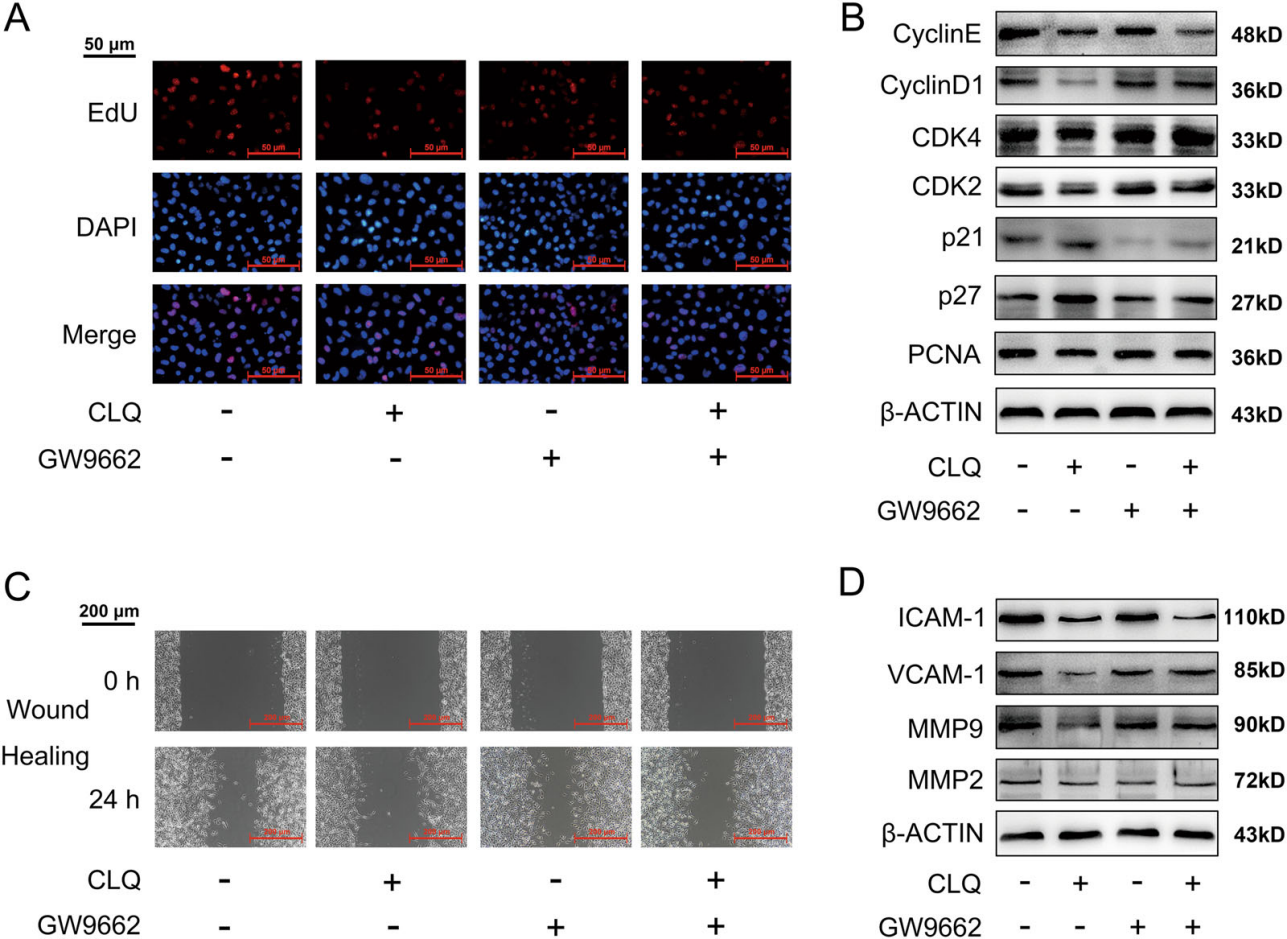
PPARγ antagonist alleviates the antimelanoma effects of CLQ in B16F10 cells. B16F10 cells were treated with 1.5 μM CLQ with/without 10 μM GW9662 for 24 h. **a** EdU incorporation assay. **b** Protein expression levels of key regulators involved in cell cycle progression. **c** Wound-healing assays. **d** Protein expression levels of ICAM-1, VCAM-1 and MMPs. All values are presented as the mean ± SD from at least three separate experiments

To explore the causal relation between PPARγ activation and CLQ pharmalogical effects, we treated B16F10 cells with GW9662 (known as a PPARγ-specific inhibitor) and PPARγ shRNA (knockdown efficiency was presented in Supplementary Fig. 7), respectively. As shown in Fig. 6a, the CLQ-induced reduction in cell proliferation was partially attenuated by GW9662. The quantitative results were presented in Supplementary Fig. 8a. Accordingly, the inhibition of the protein expression levels of PCNA, Cyclin D1 and CDK4 in CLQ-treated cells was significantly abolished, whereas the accumulation of negative cell cycle regulators, including p27 and p21, was decreased in GW9662/CLQ-cotreated cells (Fig. 6b and Supplementary Fig. 8b). For cell migration, wound-healing assay indicated that GW9662 antagonized the anti-migration properties of CLQ (Fig. 6c). The quantitative results were presented in Supplementary Fig. 8c. At the molecular level, CLQ-induced suppression of the migration-related proteins, such as VCAM-1 and MMP-9, was strongly alleviated by GW9662 (Fig. 6d and Supplementary Fig. 8d). Similar results were observed when PPARγ was specifically knocked down by shRNA, emphasizing a critical role of PPARγ in mediating the antimelanoma action of CLQ (Supplementary Fig. 9).

### PPARγ mediates the inhibitory effects of CLQ on glycolysis in B16F10 cells

**Fig. 7:**
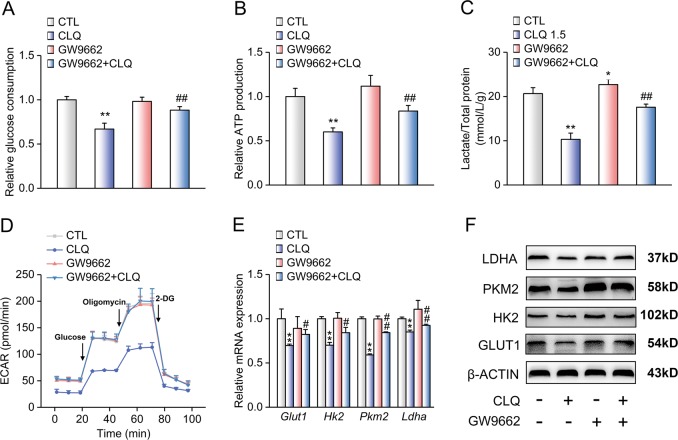
PPARγ mediates the inhibitory effects of CLQ on glycolysis in B16F10 cells. B16F10 cells were treated with 1.5 μM CLQ with/without 10 μM GW9662 for 24 h. **a** Glucose consumption. **b** Relative ATP production. **c** Lactate production. **d** ECAR. **e** RT-qPCR and (**f**) Western blot analyses of GLUT1, HK2, PKM2, LDHA expression levels. All values are presented as the mean ± SD from at least three separate experiments. *P < 0.05 and **P < 0.01 vs. CTL, ^#^*P* < 0.05 and ^##^*P* < 0.01 vs. CLQ

To determine the mediatory role of PPARγ in CLQ-suppressed glycolysis, we similarly incubated B16F10 cells with GW9662. As expected, we found that GW9662 significantly accelerated glucose consumption while increasing lactate production and ATP generation in CLQ-treated B16F10 cells (Fig. 7a–c). Consistently, ECAR analysis indicated that the CLQ-inhibited glycolytic flux was abolished in presence of GW9662 (Fig. 7d and Supplementary Fig. 10a). At the molecular level, GW9662 abrogated the CLQ-induced reduction of glycolytic genes, including HK2, PKM2 and LDHA, at both the transcriptional and translational levels (Fig. 7e, f and Supplementary Fig. 10b).

## Discussion

In our present study, we performed cellular screening with the FDA-approved drug library to identify new suppressors of melanoma. CLQ was ultimately selected for further characterization as an antimelanoma drug not only because of its strong inhibitory effects on B16F10 cell proliferation (inhibition rate > 75%) but also because its antitumor effect has never been demonstrated before. We found that CLQ decreased melanoma tumor growth and metastasis in vivo and in vitro. In addition, CLQ ameliorated the “Warburg effect” in melanoma B16F10 cells. On the other hand, PharmMapper and DRAR-CPI analyses indicated that PPARγ was the most likely molecular target in relaying CLQ signals. More importantly, the specific PPARγ inhibitor GW9662 or its shRNA partially abolished antimelanoma effects of CLQ.

As a well-established drug, CLQ is used to treat tuberculosis and exhibits powerful anti-infection and antibacterial properties^20^. A maximum tolerated dose study in mice revealed that the administration of CLQ (dosages <80 mg/kg) did not lead to any overt signs of toxicity, including abnormal behavior or other side effects^15^. Therefore, the doses we used (maximum dose 25 mg/kg) in mice are rather low and considered safe. We also evaluated key parameters involved in the assessment of liver toxicity, including the serum AST and ALT levels, liver histology and hepatocytes apoptosis. As shown in Supplementary Fig. 2, all these parameters were within their physiological ranges. Therefore, such a low dose of CLQ may dramatically decrease toxic crisis and improve patient tolerance in the future translational applications. More studies are needed to pursue the antimelanoma effects of CLQ in the clinic based on these low doses.

The overactivated growth of melanoma cells is an important event in the pathogenesis of cancer and is directly linked to the failure of clinical interventions used to treat melanoma cancer patients. In the present study, CLQ significantly decreased proliferation of melanoma cells and B16F10 xenograft tumor growth. In addition, the cell cycle is tightly related to cell proliferation and is finely controlled by multiple proteins, such as CDKs, cyclins and CDK inhibitors^21^. Our analyses showed a dramatic dose-dependent induction of the CDK inhibitors p21 and p27 and a marked reduction of CDKs (CDK2 and CDK4) and cyclins (Cyclin E and Cyclin D1) in CLQ-treated B16F10 and A375 cells. In addition, tumor metastasis represents a major complication in clinical treatment lowering the survival rate of melanoma cancer patients^22^. In our study, we found that CLQ exhibited an excellent ability to inhibit migration of melanoma cells in vitro and remarkably decreased lung metastasis of B16F10 in vivo. Additionally, the protein expression levels of metastasis-associated genes (such as MMP2/9, ICAM-1 and VCAM-1) in melanoma cells were suppressed by CLQ treatment. Taken together, these data suggest that the inhibitory effects of CLQ on melanoma cell proliferation and migration are related to the suppression of the whole cell cycle and the hallmark protein expression involved in these processes.

Cancer cell proliferation and metastasis are closely associated with changes in glucose metabolism in tumors. Cancer cells exhibit high rates of aerobic glycolysis, a phenomenon historically known as the “Warburg effect”^23,24^. Accordingly, glucose uptake in these cells is increased to ensure the energy supply to compensate for the shortage of ATP because of glycolysis, leading to an excessive generation of lactate^23,24^. At the molecular level, GLUT1 contributes to the increased glucose utilization^25^. Next, HK2 catalyzes the first step of glucose metabolism, phosphorylating glucose to glucose-6-phosphate (G6P), while PKM2 switches the G6P into the glycolytic intermediate, pyruvate, which is converted into lactate by LDHA^26^. Remarkably, the inhibition of glycolysis suppresses tumor cell proliferation and migration^27^. For example, the inhibition of HK diminishes proliferation of cancer cells by decreasing the release of signaling proteins^28^. The present study showed that CLQ exerted its antimelanoma effect by decreasing glucose consumption as well as ATP and lactate production by B16F10 cells. In addition, CLQ induced a major reduction of glycolysis in B16F10 cells. Consistently, CLQ significantly reduced the expression of glycolytic genes at both transcriptional and translational levels. To dissect the potential role of glycolysis in CLQ-induced impairment of B16F10 growth and metastasis, 2-DG, a competitive inhibitor of glucose transporters, was used to block the glycolysis in vivo and in vitro. We found that while CLQ and 2-DG alone suppressed the growth and metastasis of B16F10 cells as expected, the combination of these two reagents did not lead to a further inhibition both in vitro and in vivo, implicating that inhibition of glycolysis is potentially involved in mediating the antimelanoma actions of CLQ. Further studies would be necessary to fully elucidate the role of glycolysis in mediating CLQ’s effects under the condition of the glycolysis inhibition and enhancement. Of note, in addition to its antitumor effects in melanoma, CLQ may also have a great potential in triggering a similar reduction of glycolysis in other cancer cells. Lastly, CLQ is also a mitochondrial uncoupler that increases oxygen consumption rate (OCR) in the presence of oligomycin in mouse cardiomyocytes. Hence, we hypothesized that CLQ may switch glycolysis to OXPHOS in B16F10 cells, which should be identified in the further study.

PPARγ is a versatile transcriptional regulator involved in many pathophysiological processes, including adipocyte differentiation and glucose metabolism^29,30^. However, the role of PPARγ in cancer is controversial, with studies showing either pro- or anti-neoplastic effects^31–33^. One possible explanation of this debate is that activation of PPARγ induces growth inhibition in epithelial cancer cells, whereas it triggers tumor growth in stromal cells, indicating that the consequences of PPARγ activation for tumorigenesis are dependent on the cellular compartment^34^. It is known that melanoma originates from the skin and exhibits an epithelial-like phenotype^35^. Hence, the activation of PPARγ in B16F10 cells may lead to growth inhibition. Consistently, treatment with PPARγ agonists dramatically blocks the proliferation of melanoma cells^36^. In the present study, we found that CLQ activated the *PPARγ* transcription and increased PPARγ nuclear contents. Also, the extents of increases in PPARγ expression and nuclear fractions are quite comparable. These data suggest that CLQ increases PPARγ activity initially at the transcriptional level, and the increase of PPARγ nuclear contents is only a consequent event. Moreover, the PPARγ-specific antagonist GW9662 or PPARγ shRNA partially abolished the antimelanoma effects of CLQ. These results indicated that PPARγ might serve as a potential molecular target of CLQ. On the other hand, CLQ could enhance the p53 activity to switch its pro-apoptotic ability into a protective response including activation of the p21 expression^15^. In our study, we found that CLQ increases the protein expression levels of p21 but does not alter p53 levels, indicating that the inhibitory effects of CLQ on melanoma are p53-independent.

PPARγ agonists, such as rosiglitazone and pioglitazone, have been widely used in the clinical treatment of various diseases, including type 2 diabetes^37^. Although these thiazolidinediones (TZDs) exhibit satisfactory effects on improving insulin sensitivity and hyperglycemia, most of them have detrimental side effects^38^. For example, troglitazone was withdrawn because of liver toxicity^39^. In addition, administration of rosiglitazone is tightly correlated with an increased risk of cardiovascular diseases in patients, while pioglitazone has been associated with an increased fracture risk^40,41^. These limitations aroused substantial concerns and significantly dampened TZD’s future in many countries. Therefore, it is critical to develop TZD substitutes or non-TZD selective PPARγ agonists for improved therapies of diseases associated with PPARγ activity. In the present study, we found that CLQ increased the *PPARγ* transcription and that such activation did not cause any liver toxicity in mice. Hence, as an FDA-approved clinical drug, CLQ may be a new non-TZD selective PPARγ agonist and further evidence should be provided to establish whether CLQ performs a similar function in the treatment of metabolic diseases, including insulin resistance and hyperglycemia.

**Fig. 8:**
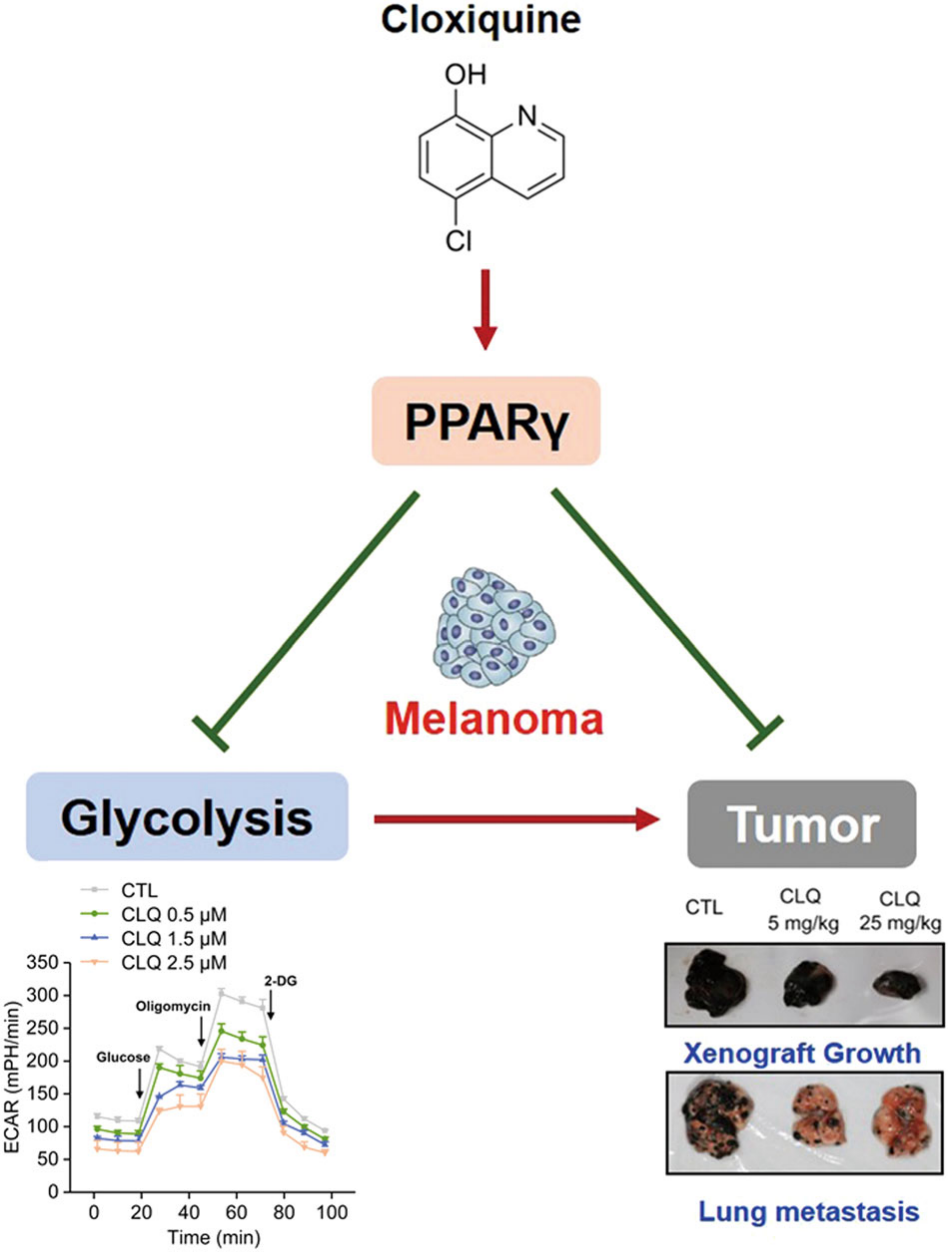
**CLQ effectively suppresses the malignant phenotypes of melanoma through activation of PPARγ**

In conclusion, our findings demonstrated the potential use of CLQ in reducing glycolysis and inhibiting proliferation and metastasis of melanoma cells. Mechanistically, PPARγ plays a nonredundant role in mediating these beneficial effects (Fig. 8). These results imply that in addition to its antituberculosis use, CLQ is a promising candidate in the therapy of melanoma cancer progression.

## Materials and methods

### Cell culture

All cell lines were obtained from the American Type Culture Collection (ATCC) and grown at 37 ℃ in 5% CO_2_-95% air. B16F10, A375 and PIG1 cells were cultured in DMEM supplemented with 10% fetal bovine serum (FBS, Sciencell Research, Carlsbad, CA, USA) and 1% antibiotics (penicillin and streptomycin). Melan-A cells were grown in RPMI-1640 medium containing 10% FBS and 1% antibiotics.

### Drug screening assay

CCK-8 assay was used to screen functional drugs that could suppress the proliferation of melanoma cells. In brief, 5 × 10^3^ cells were seeded into each well of a 96-well plate and cultured at 37 °C overnight. After synchronization with serum-free DMEM, cells were transferred into 100 μL of serum-free DMEM containing either 10 μM drugs (1430 small compounds) or vehicle (0.1% DMSO) and incubated for another 24 h. Then, 10 μL of WST-8 reagent (Jiancheng, Nanjing, Jiangsu, China) was added to each well and incubated at 37 °C for 2 h. Finally, a microplate reader was used to measure the absorbance at 450 nm. An inhibition efficiency of cell viability more than 75% were considered significant.

### Morphometric analysis

All cell lines were treated with CLQ for 24 h and then fixed in 4% paraformaldehyde solution for 30 min. Cell morphologies were photographed with a Nikon microscope (ECLIPSE, Ts2R-FL, Tokyo, Japan).

### Proliferation and migration assays

Cell proliferation was analyzed by using an EdU incorporation assay, while cell migration was detected by using wound healing and transwell chamber assays. These experiments were performed as described previously^17^.

### RT-qPCR and Western blot

Total RNA from B16F10 cells were extracted using Trizol reagent (Invitrogen, Carlsbad, CA, USA). For mRNA detection, 1 μg total RNA was reverse-transcribed into complementary DNA. Real-time PCR amplification was performed using SYBR premix Ex Taq (Vazyme, Nanjing, Jiangsu, China) and the LightCycler® 480 System (Roche, Basal, Switzerland). The primers for mouse 36B4 were included for normalization. A complete list of PCR primers is shown in Supplementary Table 4. For protein analysis, cells were lysed in RIPA buffer. The protein concentration was quantified with a BCA protein quantification kit (Jiancheng, Nanjing, Jiangsu, China). Equal amounts of protein were loaded and separated by 10% SDS-PAGE and then transferred onto PVDF membranes (Millipore, Bedford, MA, USA). The membranes were incubated overnight with appropriate primary antibodies. HRP-conjugated secondary antibodies were then applied to bind and visualize the primary antibodies. Quantitative analysis was performed by NIH ImageJ 1.32j software. To detect the translocation of PPARγ protein to the nuclei, we extracted nuclear protein using a commercial kit (Shengxing, Shanghai, China) and then performed Western blot analysis. Histone H3 was used as the loading control. Antibodies against Cyclin E (Cat. No. 4129; 1:1000 dilution), PCNA (Cat. No. 2586; 1:1000 dilution), CDK2 (Cat. No. 2546; 1:1000 dilution), Cyclin D1 (Cat. No. 2922; 1:1000 dilution), p27 (Cat. No. 3686; 1:1000 dilution) and PPARγ (Cat. No. 2443 S; 1:1000 dilution) were purchased from Cell Signaling Technology (Danvers, MA, USA). Antibodies against CDK4 (Cat. No. 610147; 1:1000 dilution) and p21 (Cat. No. 556430; 1:1000 dilution) were purchased from BD Bioscience (San Jose, CA, USA). Antibodies against ICAM-1 (Cat. No. sc-1511; 1:500 dilution), VCAM-1 (Cat. No. sc-1504; 1:500 dilution), MMP-2 (Cat. No. sc-10736; 1:500 dilution) and MMP-9 (Cat. No. sc-6840; 1:500 dilution) were purchased from Santa Cruz (Dallas, TX, USA). Antibodies against LDHA (Cat. No. 19987; 1:1000 dilution), PKM2 (Cat. No. 15822; 1:1000 dilution), HK2 (Cat. No. 13938; 1:1000 dilution), GLUT1 (Cat. No. 21829; 1:1000 dilution) and p53 (Cat. No. 10442; 1:1000 dilution) were purchased from Proteintech (Chicago, IL, USA). Antibodies against Histone H3 (Cat. No. BS1174; 1:1000 dilution) and β-ACTIN (Cat. No. BS6007MH; 1:1000 dilution) were purchased from Bioworld Technology (Nanjing, Jiangsu, China).

### Animals

All animal procedures in this investigation conform to the Guide for the Care and Use of Laboratory Animals published by the US National Institutes of Health (NIH publication No. 85–23, revised 1996) and the approved regulations set by the Laboratory Animal Care Committee at China Pharmaceutical University (Permit number SYXK-2016–0011). All mice were maintained in a 12 h light/12 h dark cycle and a temperature- and humidity-controlled environment. To detect the effect of tumor growth in vivo, mouse B16F10 cells (1 × 10^6^ cells per mouse) were injected subcutaneously into the right flanks of nude mice (Model Animal Research Center of Nanjing University, Nanjing, Jiangsu, China). Tumors were allowed to grow for seven days, and then mice were administered vehicle (olive, intraperitoneally (*i.p*.), daily) or CLQ (5 mg/kg, 25 mg/kg, *i.p*., daily) for eight days. The dose of CLQ was chosen based on a previous study showing that this CLQ dose functionally protected from radiation-induced gastrointestinal death in mice^15^. For the metastasis analysis, mouse B16F10 cells (1.5 × 10^5^ cells per mouse) were injected intravenously. Seven days later, mice were randomly divided into three groups and were administered vehicle (olive, *i.p*., daily) or CLQ (5 mg/kg, 25 mg/kg, *i.p*., daily) for fourteen days. Then, mice were euthanized, their lungs were removed and fixed, and the number of macroscopic black metastases on the surfaces of the lungs were enumerated. To detect whether glycolysis mediates the antimelanoma effects of CLQ, 2-DG was *i.p*. injected into mice at a dose of 500 mg/kg body weight once every other day in addition to CLQ (25 mg/kg, *i.p*., daily) administration.

### IHC and TUNEL assays

All specimens were isolated, fixed in 4% paraformaldehyde solution for 24 h in situ, processed for paraffin embedding, and cut into 4 µm transverse sections for routine H&E staining. For IHC analysis, tumor sections were incubated with antibody against mouse Ki-67 (Cat. No. 12202; 1:200 dilution, Cell Signaling Technology) at 4 °C overnight for the later immunostaining by using diaminobenzidine (DAB). For TUNEL analysis, liver sections were incubated with TUNEL labeling (marked with Green fluorescence) mix at 37 °C for 60 min, and then double stained with DAPI (marked with Blue fluorescence, Sigma, USA). The sections were photographed with a Nikon fluorescence microscope (ECLIPSE, Ts2R-FL, Tokyo, Japan). The slides were scanned by a Pannoramic Flash 250 scanner (Perkin Elmer, Waltham, MA, USA) and viewed by the Pannoramic viewer software program (3D Histech, Waltham, MA, USA). Quantitative analysis was performed by NIH ImageJ 1.32j software.

### Serological analysis

Blood samples were collected in nonheparinized tubes and centrifuged at 4000 r.p.m. for 10 min at 4 °C. The serum levels of ALT and AST were determined spectrophotometrically using commercial kits (Jiancheng Institute of Biotechnology, Nanjing, Jiangsu, China).

### Glucose consumption, ATP and lactate production

Mouse B16F10 cells were treated with indicated doses of CLQ with/without GW9662 (10 μM, PPARγ antagonist) for 24 h. The concentration of glucose in culture medium was measured by the Glucose Test Kit (Rongsheng Biotechnology, Shanghai, China). The intracellular concentrations of lactate and ATP were determined by using commercial kits (Sigma, St Louis, MO, USA for lactate, and Promega, Madison, WI, USA for ATP), according to the manufacturer’s instructions.

### ECAR assay

The ECAR was analyzed using a Seahorse XF24 Extracellular Flux Analyzer (Seahorse Bioscience, North Billerica, MA, USA). In brief, 2 × 10^5^ cells were seeded into each well of a Seahorse XF24 cell culture microplate. On the next day, the cells were treated with/without CLQ at indicated doses for 24 h, and GW9662 was added when necessary. Then, cells were switched to a basal glucose-free medium for 1 h prior to measurement. A default standard glycolysis stress-test program was performed. During measurement, glucose (final concentration 10 mM), oligomycin (final concentration 1 μM) and 2-DG (final concentration 100 mM) were sequentially injected into each well at the indicated time points. Data were derived from Seahorse XF24 Wave software and displayed in pmol/minute.

### Reverse pharmacophore screening

To predict the potential molecular target of CLQ, we performed PharmMapper (http://lilab.ecust.edu.cn/pharmmapper/) and DRAR-CPI (http://cpi.bio-x.cn/drar/) analyses. PharmMapper is a web-based tool that predicts the binding of a small molecule to potential targets via a “reverse” pharmacophore mapping approach^42^. All the predicted targets were screened and sorted by Normalized Fit-score in a descending order. DRAR-CPI is a server that is used for repositioning drugs based on the analysis of the chemical-protein interactome^43^. All DRAR-CPI-calculated results were ordered by the default index Z’-score in ascending order. After prediction, the top 10 candidates were selected and clustered for their potential targets.

### ICC assay

To analyze the PPARγ intracellular localization, mouse B16F10 cells were treated with or without CLQ (1.5 μM, an effective dose in inhibiting proliferation, migration, and glycolysis in B16F10 cells) for 24 h. After that, cells were fixed with ice-cold 4% paraformaldehyde for 30 min, followed by blocking in 5% goat serum for 1 h, and incubation with rabbit polyclonal anti-PPARγ antibody overnight at 4 °C. After repeated washing, the cells were probed with secondary antibodies conjugated to Alexa Fluor 488 anti-Rabbit IgG (Cat. No. 4408 S, 1:500 dilution, Cell Signaling Technology) for 1 h at room temperature. Nuclei were identified with DAPI. The sections were photographed with a Nikon fluorescence microscope (ECLIPSE, Ts2R-FL, Tokyo, Japan).

### PPARγ shRNA transfection

Mouse PPARγ shRNA (5′-CCATCCGATTGAAGCTTATTTATGA-3′) and non-specific shRNA (Scramble shRNA, 5′-TTCTCCGAACGTGTCACGTAA-3′) were designed and synthesized by GenePharma (Shanghai, China). To knockdown the expression of PPARγ in B16F10 cells, the cells were transfected with scramble shRNA or PPARγ shRNA 24 h in advance of indicated treatments. Cell transfection were performed by using Lipofectamine 3000 reagent (Invitrogen, Carlsbad, CA, USA) according to the manufacturer’s instructions.

### Statistical analysis

Statistical analysis was performed by using the Origin 8 software (version 8.6, OriginLab Corporation, USA). Groups of data were presented as the mean ± SD (standard deviation). One-way ANOVA followed by Fisher’s LSD post hoc test were performed to analyze the data. A value of *P* < 0.05 was considered as statistically significant.

## Supplementary information

Supplementary Figure Legends

Supplementary Tables

Supplementary Figure 1

Supplementary Figure 2

Supplementary Figure 3

Supplementary Figure 4

Supplementary Figure 5

Supplementary Figure 6

Supplementary Figure 7

Supplementary Figure 8

Supplementary Figure 9

Supplementary Figure 10
