# Supplementary material for: Cloxiquine, a traditional antituberculosis agent, suppresses the growth and metastasis of melanoma cells through activation of PPARγ
Source: Cell Death Dis. 2019 May 28;10(6):404. doi: 10.1038/s41419-019-1644-8 (PMC6538643; doi:10.1038/s41419-019-1644-8)
Supplement: Supplementary file 1 — Supplementary Figure Legends [file 41419_2019_1644_MOESM1_ESM.doc]

**Supplementary Figure Legends**

**Figure S1. CLQ inhibits the proliferation of melanoma cells.** Quantification of the EdU incorporation assay presented in **Fig. 2a.** All values are presented as the mean ± SD. *^**^P* < 0.01 *vs.* CTL.

**Figure S2. CLQ exhibits modest hepatotoxicity in mice. a** Serum ALT and AST. All values are presented as the mean ± SD. n=5 for each group. **b** H&E and **c** TUNEL staining of liver sections from mice treated with/without CLQ.

**Figure S3. CLQ inhibits the migration of melanoma cells.** **a** Quantification of the transwell chamber assay presented in **Fig. 3a**. **b** Quantification of the wound healing assay presented in **Fig. 3a**. All values are presented as the mean ± SD from at least three separate experiments. *^*^P* < 0.05 and *^**^P* < 0.01 *vs.* CTL.

**Figure S4. CLQ suppresses the glycolysis in mouse B16F10 cells.** **a** Key parameters of glycolysis tested in **Fig. 4d**. **b** Quantification for the immunoblots in **Fig. 4f**. All values are presented as the mean ± SD from at least three separate experiments. *^*^P* < 0.05 and *^**^P* < 0.01 *vs.* CTL.

**Figure S5. Combination of 2-DG and CLQ does not exhibit synergistic antimelanoma effects.** Mouse B16F10 cells were treated with 1.5 μM CLQ with/without 2-DG (5 mM) for 24 h. **a** EdU incorporation and wound-healing assays. **b** Quantification of the EdU incorporation and wound-healing assays presented in panel **a**. **c** Protein expression levels of key regulators involved in cell cycle progression and migration. **d** Quantification for the immunoblots in panel **c**. All values are presented as the mean ± SD from at least three separate experiments. *^*^P* < 0.05 and *^**^P* < 0.01 *vs.* CTL. Xenograft tumor model and Lung metastatic model were constructed similarly within **Fig. 2** and **Fig.3**, respectively. Note that these mice were administered with 2-DG (500mg/kg, *i.p.*, once every other day) in addition to the CLQ treatment. n=5 for each group. For tumor growth analysis, **e** Representative tumor images. **f** Tumor weights. **g** H&E staining and immunohistochemistry analysis of Ki-67 from tumor sections. For lung metastatic analysis, **h** Macroscopic images, **i** Statistical analyses and **j** H&E staining of lung metastatic nodules. All values are presented as the mean ± SD from at least three separate experiments. *^**^P* < 0.01 *vs.* CTL.

**Figure S6.** **CLQ modestly changed p53 protein expression and increased PPARγ expression. a** Western blot analysis of the p53 protein expression level. **b** and **c** Quantification for the immunoblots in **Fig. 6c** and **Fig. 6d**. All values are presented as the mean ± SD from at least three separate experiments. *^*^P* < 0.05 and *^**^P* < 0.01 *vs.* CTL.

**Figure S7. Knockdown efficiency of PPARγ shRNA in B16F10 cells.** RT-qPCR analysis of *Pparγ* mRNA expression level in B16F10 cells transfected with either Scramble shRNA or PPARγ shRNA. All values are presented as the mean ± SD from at least three separate experiments. *^**^P* < 0.01 *vs.* Scramble shRNA.

**Figure S8.** **PPARγ antagonist alleviates the antimelanoma effects of CLQ in B16F10 cells. a** Quantification of the EdU incorporation assay presented in **Fig.7a. b** Quantification for the immunoblots in **Fig. 7b**. **c** Quantification of the wound healing assay presented in **Fig. 7c**. **d** Quantification for the immunoblots in **Fig. 7d**. All values are presented as the mean ± SD from at least three separate experiments. *^*^P* < 0.05 and *^**^P* < 0.01 *vs.* CTL, *^##^P* < 0.01 *vs.* CLQ.

**Figure S9.** **Knockdown of PPARγ abrogates the antimelanoma effects of CLQ in B16F10 cells****.** B16F10 cells were transfected with either scramble shRNA or PPARγ shRNA for 24 h in advance of the CLQ treatment. **a** EdU incorporation assay. **b** Wound-healing assays. **c** Protein expression levels of key regulators involved in cell cycle progression. **d** Protein expression levels of ICAM-1, VCAM-1 and MMPs. **e** Quantification for the immunoblots in panel **c and d.** All values are presented as the mean ± SD from at least three separate experiments. *^**^P* < 0.01 *vs.* CTL, *^##^P* < 0.01 *vs.* CLQ.

**Figure S10.** **PPARγ mediates the inhibitory effects of CLQ on glycolysis in B16F10 cells.** **a** Key parameters of glycolysis tested in **Fig. 8d**. **b** Quantification for the immunoblots in **Fig. 8f**. All values are presented as the mean ± SD from at least three separate experiments. *^**^P* < 0.01 *vs.* CTL, *^#^P* < 0.05 and *^##^P* < 0.01 *vs.* CLQ.
