# Supplementary material for: Cloxiquine, a traditional antituberculosis agent, suppresses the growth and metastasis of melanoma cells through activation of PPARγ
Source: Cell Death Dis. 2019 May 28;10(6):404. doi: 10.1038/s41419-019-1644-8 (PMC6538643; doi:10.1038/s41419-019-1644-8)
Supplement: Supplementary file 2 — Supplementary Tables [file 41419_2019_1644_MOESM2_ESM.doc]

**Supplementary Tables**

**Table S1. Potential anti-melanoma drugs**

| **No.** | **Drugs** | **Inhibition**  **ratio (B16F10/A375)** | **Targets** | **Roles in tumorigenesis** |
| --- | --- | --- | --- | --- |
| 1 | Foretinib | 92.07/42.55 | c-Met  VEGFR | Foretinib demonstrates anti-tumor activity and improves overall survival in preclinical models of hepatocellular carcinoma. Angiogenesis, 2012, 15(1): 59-70. |
| 2 | Doxorubicin | 89.17/67.62 | Autophagy,  Topoisomerase | Effects of doxorubicin cancer therapy on autophagy and the ubiquitin-proteasome system in long-term cultured adult rat cardiomyocytes. Cell and tissue research, 2012, 350(2): 361-372. |
| 3 | CUDC-101 | 90.91/35.06 | EGFR  HDAC  HER | Discovery of 7-(4-(3-ethynylphenylamino)-7-methoxyquinazolin-6-yloxy)-N-hydroxyheptanamide (CUDC-101) as a potent multi-acting HDAC, EGFR, and HER2 inhibitor for the treatment of cancer. Journal of medicinal chemistry, 2010, 53(5): 2000-2009. |
| 4 | Nitazoxanide | 84.55/25.36 | Others | Thiazolides inhibit growth and induce glutathione‐S‐transferase Pi (GSTP1)‐dependent cell death in human colon cancer cells. International journal of cancer, 2008, 123(8): 1797-1806. |
| 5 | Silmitasertib (CX-4945) | 83.15/87.68 | Casein Kinase | The casein kinase 2 inhibitor, CX-4945, as an anti-cancer drug in treatment of human hematological malignancies. Frontiers in pharmacology, 2015, 6: 70. |
| 6 | Toltrazuril | 77.84/55.56 | Antifection | Chemotherapy with fumagillin and toltrazuril against kidney enlargement disease of goldfish caused by the myxosporean Hoferellus carassii. Fish Pathology, 1990, 25(3): 157-163. |
| 7 | Triclabendazole | 84.13/70.90 | Microtubule Associated | Cervical tumor caused by the sexually mature stage of Fasciola hepatica. Transactions of the Royal Society of Tropical Medicine and Hygiene, 2009, 103(3): 318-320. |
| 8 | Benzethonium Chloride | 96.16/90.07 | AChR | Benzethonium chloride: a novel anticancer agent identified by using a cell-based small-molecule screen. Clinical Cancer Research, 2006, 12(18): 5557-5569 |
| 9 | Closantel | 92.05/35.73 | Antifection | Closantel suppresses angiogenesis and cancer growth in zebrafish models. Assay and drug development technologies, 2016, 14(5): 282-290. |
| 10 | Thioridazine HCl | 89.08/92.95 | Others | Thioridazine induces apoptosis by targeting the PI3K/Akt/mTOR pathway in cervical and endometrial cancer cells. Apoptosis, 2012, 17(9): 989-997. |
| 11 | Cloxiquine | 76.53/49.22 | Antifection | Unknown |
| 12 | Clofoctol | 79.17/91.05 | Others | Identification of an old antibiotic clofoctol as a novel activator of unfolded protein response pathways and an inhibitor of prostate cancer. British journal of pharmacology, 2014, 171(19): 4478-4489. |
| 13 | Bithionol | 90.95/61.97 | Camp | Assessment of the anti-tumor potential of Bithionol in vivo using a xenograft model of ovarian cancer. Anti-cancer drugs, 2016, 27(6): 547. |
| 14 | Hexachlorophene | 89.98/58.33 | Potassium Channel | Hexachlorophene inhibits Wnt/β-catenin pathway by promoting Siah-mediated β-catenin degradation. Molecular pharmacology, 2006 |
| 15 | Nitroxoline | 75.97/44.79 | Topoisomerase | Effect of nitroxoline on angiogenesis and growth of human bladder cancer. Journal of the National Cancer Institute, 2010, 102(24): 1855-1873. |
| 16 | Afuresertib (GSK2110183) | 83.29/25.27 | AKT | Phase I study of the MEK inhibitor trametinib in combination with the AKT inhibitor afuresertib in patients with solid tumors and multiple myeloma. Cancer chemotherapy and pharmacology, 2015, 75(1): 183-189. |
| 17 | Entrectinib (RXDX-101) | 95.74/87.80 | Trk receptor  ALK | Durable clinical response to entrectinib in NTRK1-rearranged non-small cell lung cancer. Journal of Thoracic Oncology, 2015, 10(12): 1670-1674. |
| 18 | Sorafenib | 89.49/84.33 | PDGFR  Raf  VEGFR | Discovery and development of sorafenib: a multikinase inhibitor for treating cancer. Nature reviews Drug discovery, 2006, 5(10): 835. |
| 19 | Cobimetinib (GDC-0973, RG7420) | 96.14/75.48 | MEK | Combined vemurafenib and cobimetinib in BRAF-mutated melanoma. New England Journal of Medicine, 2014, 371(20): 1867-1876. |

**Table S2. List of the Top 10 potential CLQ targets analyzed by Pharmmapper**

| **No.** | **Putative target** | **Function** | **Normalized Fit-score** |
| --- | --- | --- | --- |
| 1 | Glutathione S-transferase P | Conjugation of reduced glutathione to hydrophobic electrophiles | 0.9161 |
| 2 | Peptidyl-prolyl cis-trans isomerase A | Posttranslational modification, protein turnover, chaperones | 0.8188 |
| 3 | Phenylethanolamine N-methyltransferase | converts noradrenaline to adrenaline | 0.7319 |
| 4 | Caspase-7 | Responsible for apoptosis execution | 0.7158 |
| 5 | Serine/threonine-protein kinase Chk1 | Required for checkpoint mediated cell cycle arrest in response to DNA damage or the presence of unreplicated DNA | 0.7126 |
| 6 | **Peroxisome proliferator-activated receptor γ** | The key regulator of the peroxisomal β-oxidation pathway of fatty acids, adipocyte differentiation and glucose homeostasis | 0.7059 |
| 7 | Liver carboxylesterase 1 | Involved in the detoxification of xenobiotics and in the activation of ester and amide prodrugs | 0.7051 |
| 8 | Mitogen-activated protein kinase 14 | Involved in MAP kinase activity | 0.6973 |
| 9 | Epidermal growth factor receptor | Involved in the control of cell growth and differentiation. | 0.6854 |
| 10 | Phenylalanine-4-hydroxylase | Unknown | 0.6741 |

**Table S3. List of the Top 10 potential CLQ targets analyzed by DRAR-CPI**

| **No.** | **Putative target** | **Function** | **Z'-score** |
| --- | --- | --- | --- |
| 1 | Macrophage migration inhibitory factor | Pro-inflammatory cytokine | -2.6813 |
| 2 | Endothelial protein C receptor | Binds activated protein C to control the blood coagulation | -2.56426 |
| 3 | Tryptophan 5-hydroxylase 1 | Unknown | -2.51546 |
| 4 | Coagulation factor IX | Vitamin K-dependent plasma protein participating in the intrinsic pathway of blood coagulation | -2.46497 |
| 5 | Aldose reductase | Catalyzes the NADPH-dependent reduction of carbonyl-containing compounds to their corresponding alcohols | -2.4289 |
| 6 | Pyruvate dehydrogenase E1 component subunit beta, mitochondrial | Catalyzes the overall conversion of pyruvate to acetyl-CoA and CO_2_ | -2.24774 |
| 7 | **Peroxisome proliferator-activated receptor γ** | The key regulator of the peroxisomal β-oxidation pathway of fatty acids, adipocyte differentiation and glucose homeostasis | -2.14399 |
| 8 | Glutathione S-transferase Mu 4 | Conjugation of reduced glutathione to hydrophobic electrophiles | -1.87912 |
| 9 | Casein kinase II subunit alpha | Participates in Wnt signaling  Phosphorylates 'Ser-392' of p53/TP53 following UV irradiation. | -1.85779 |
| 10 | Plasminogen activator inhibitor 1 | A major control point in the regulation of fibrinolysis | -1.82574 |

**Table S4. Lists of primer sequences for qPCR analysis**

| **Mouse Genes** | **Primer Sequences (5’-3’)** |
| --- | --- |
| *36B4* Forward | GAAACTGCTGCCTCACATCCG |
| *36B4* Reverse | GCTGGCACAGTGACCTCACACG |
| *Glut1* Forwards | GCTGTGCTTATGGGCTTCTC |
| *Glut1* Reverse | AGAGGCCACAAGTCTGCATT |
| *Hk2* Forward | GGGACGACGGTACACTCAAT |
| *Hk2* Reverse | GCCAGTGGTAAGGAGCTCTG |
| *Pkm2* Forward | CTGCAGGTGAAGGAGAAAGG |
| *Pkm2* Reverse | AGATGCAAACACCATGTCCA |
| *Ldha* Forward | CCGTTACCTGATGGGAGAGA |
| *Ldha* Reverse | GTAGGCACTGTCCACCACCT |
| *Pparγ* Forward | TTTTCAAGGGTGCCAGTTTC |
| *Pparγ* Reverse | AATCCTTGGCCCTCTGAGAT |
