# Supplementary figures and images for: Cloxiquine, a traditional antituberculosis agent, suppresses the growth and metastasis of melanoma cells through activation of PPARγ
Source: Cell Death Dis. 2019 May 28;10(6):404. doi: 10.1038/s41419-019-1644-8 (PMC6538643; doi:10.1038/s41419-019-1644-8)

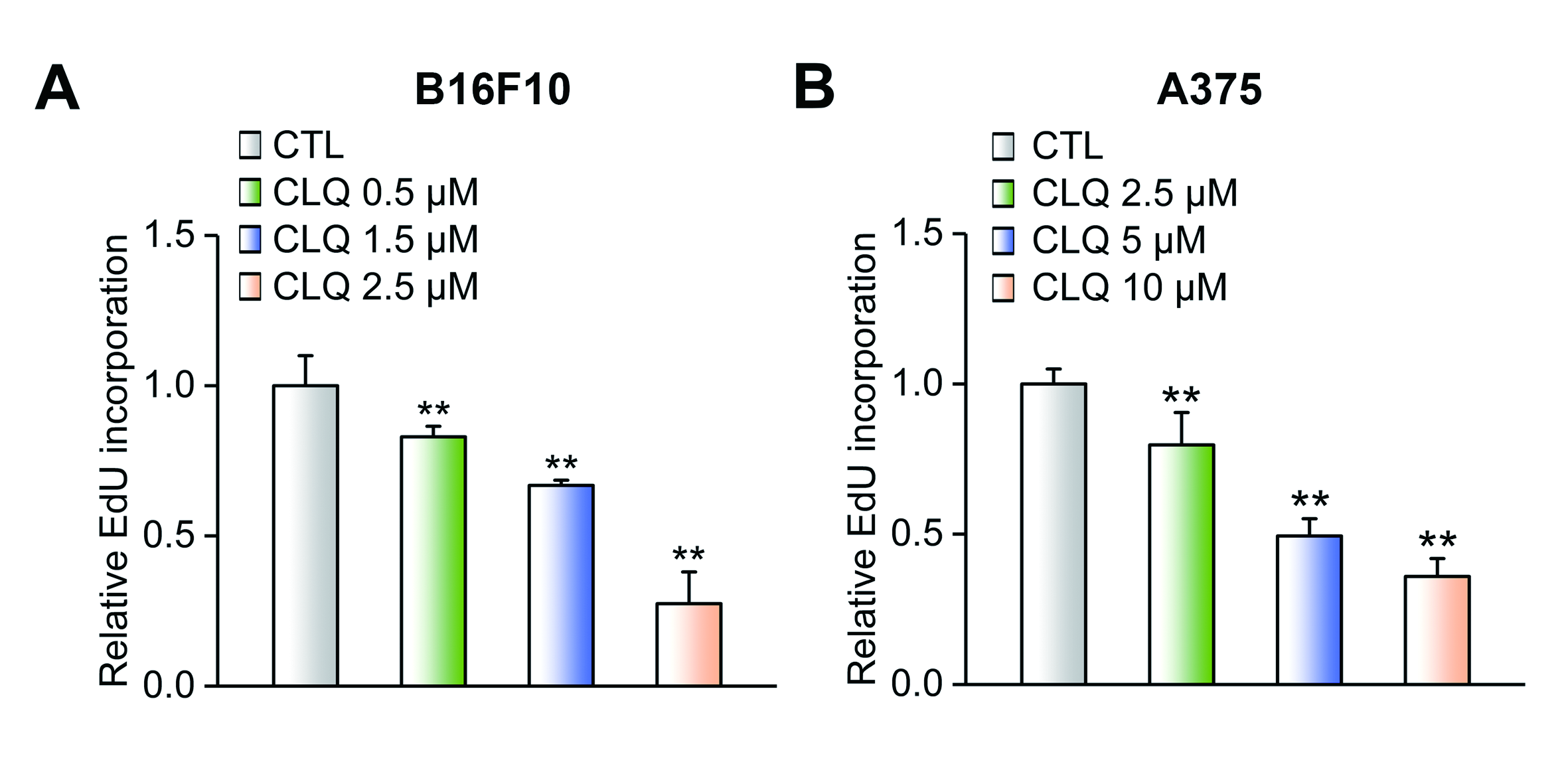

Supplement: Supplementary file 3 — Supplementary Figure 1 [file 41419_2019_1644_MOESM3_ESM.tif]

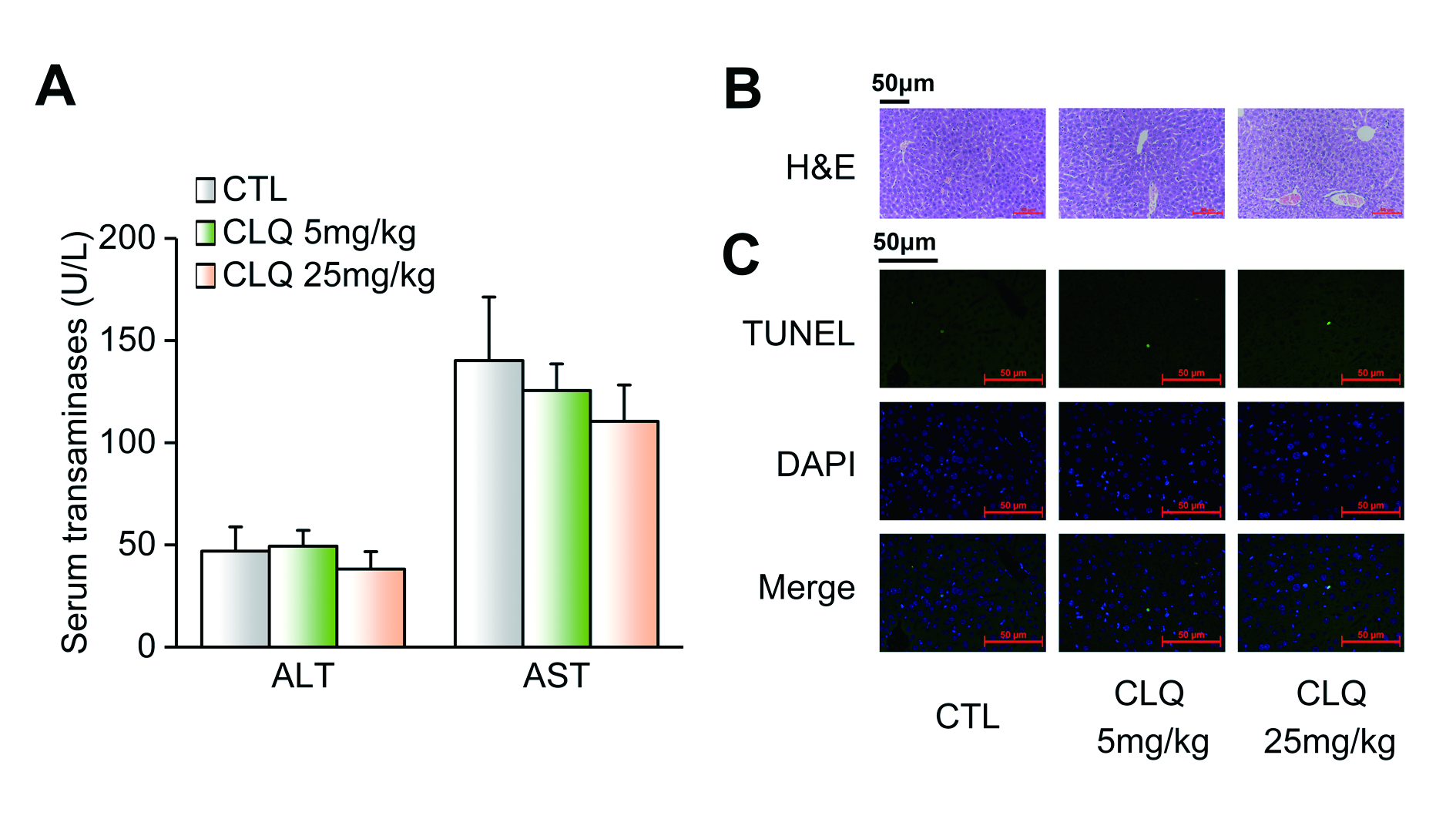

Supplement: Supplementary file 4 — Supplementary Figure 2 [file 41419_2019_1644_MOESM4_ESM.tif]

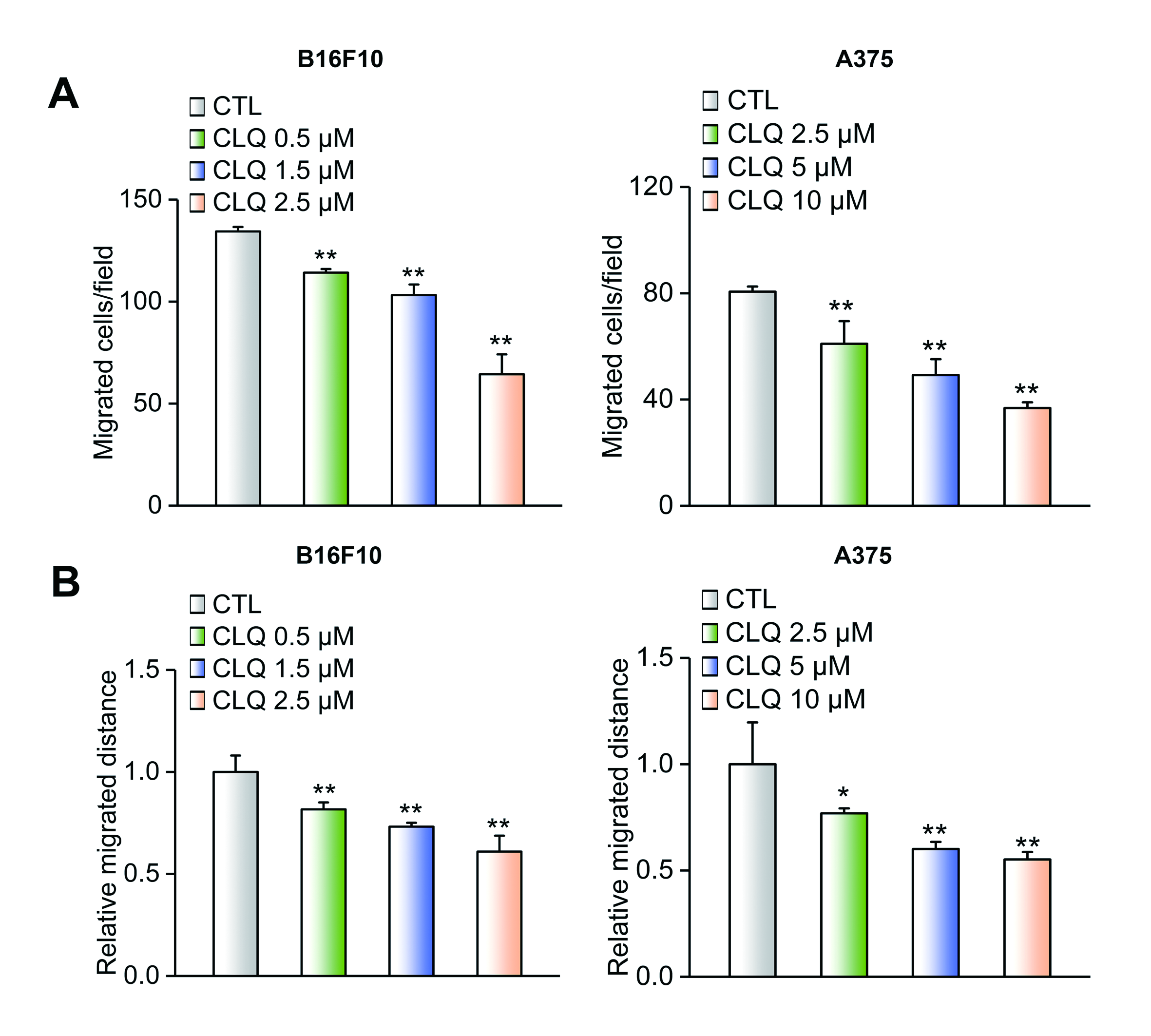

Supplement: Supplementary file 5 — Supplementary Figure 3 [file 41419_2019_1644_MOESM5_ESM.tif]

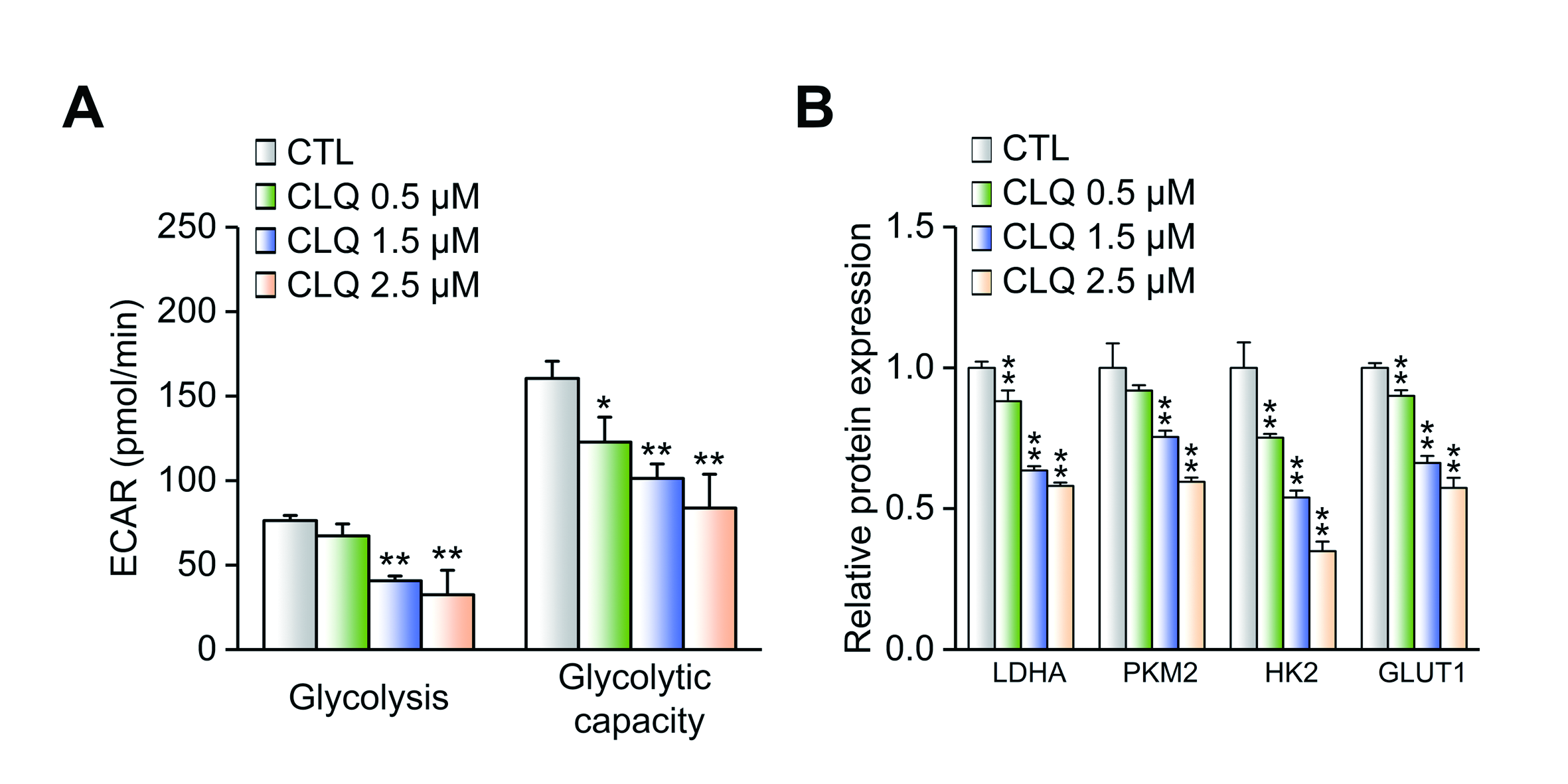

Supplement: Supplementary file 6 — Supplementary Figure 4 [file 41419_2019_1644_MOESM6_ESM.tif]

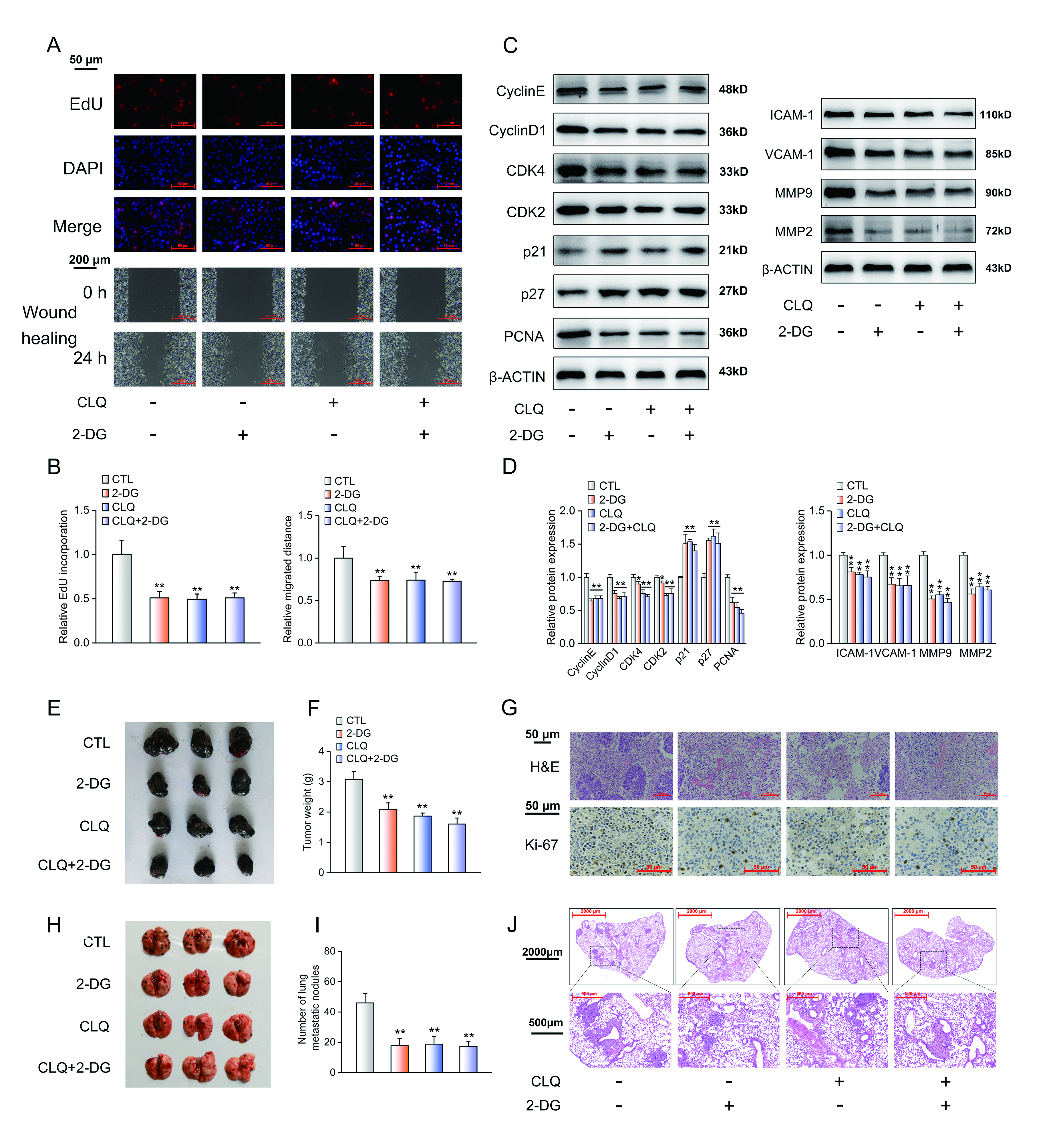

Supplement: Supplementary file 7 — Supplementary Figure 5 [file 41419_2019_1644_MOESM7_ESM.tif]

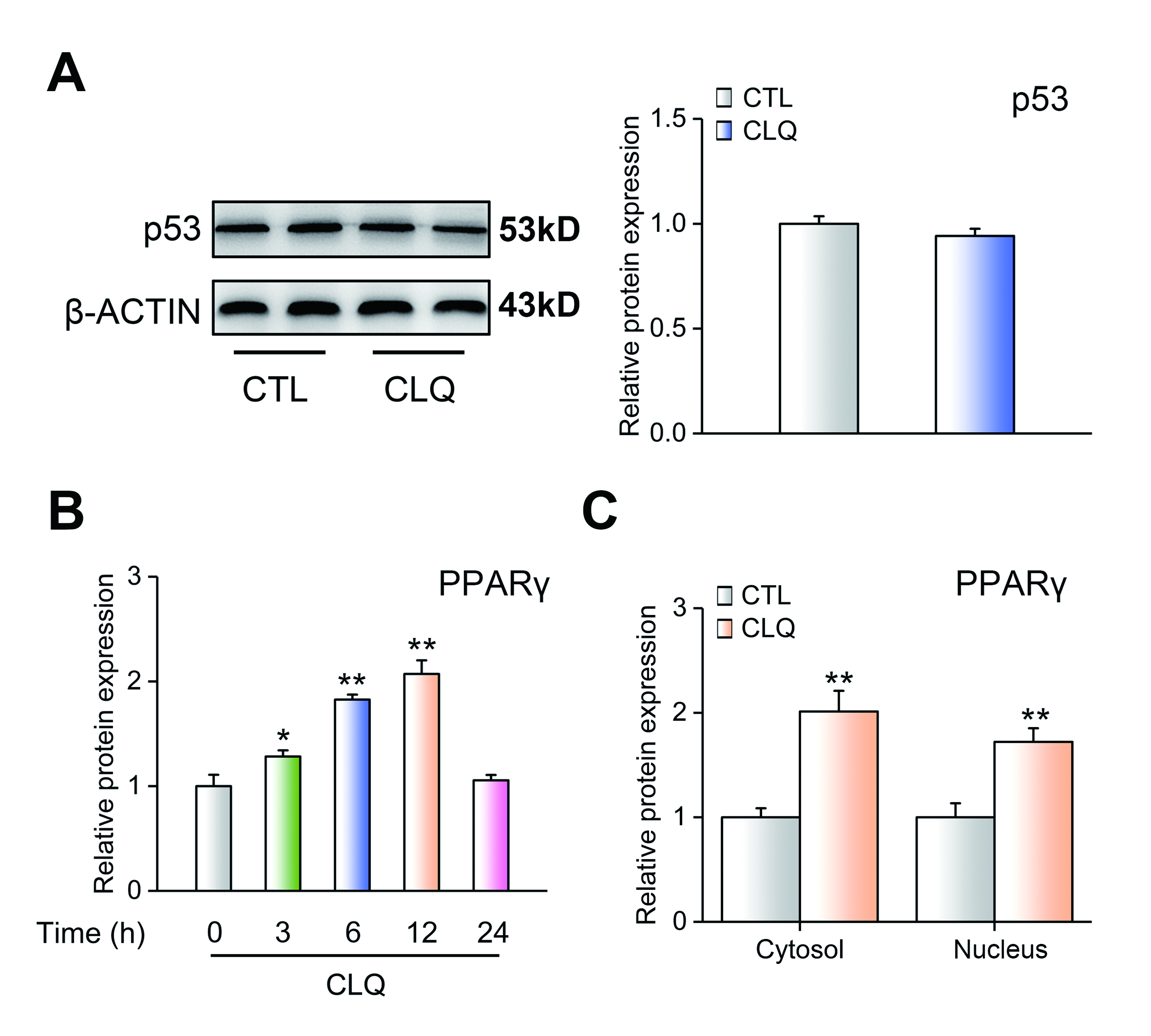

Supplement: Supplementary file 8 — Supplementary Figure 6 [file 41419_2019_1644_MOESM8_ESM.tif]

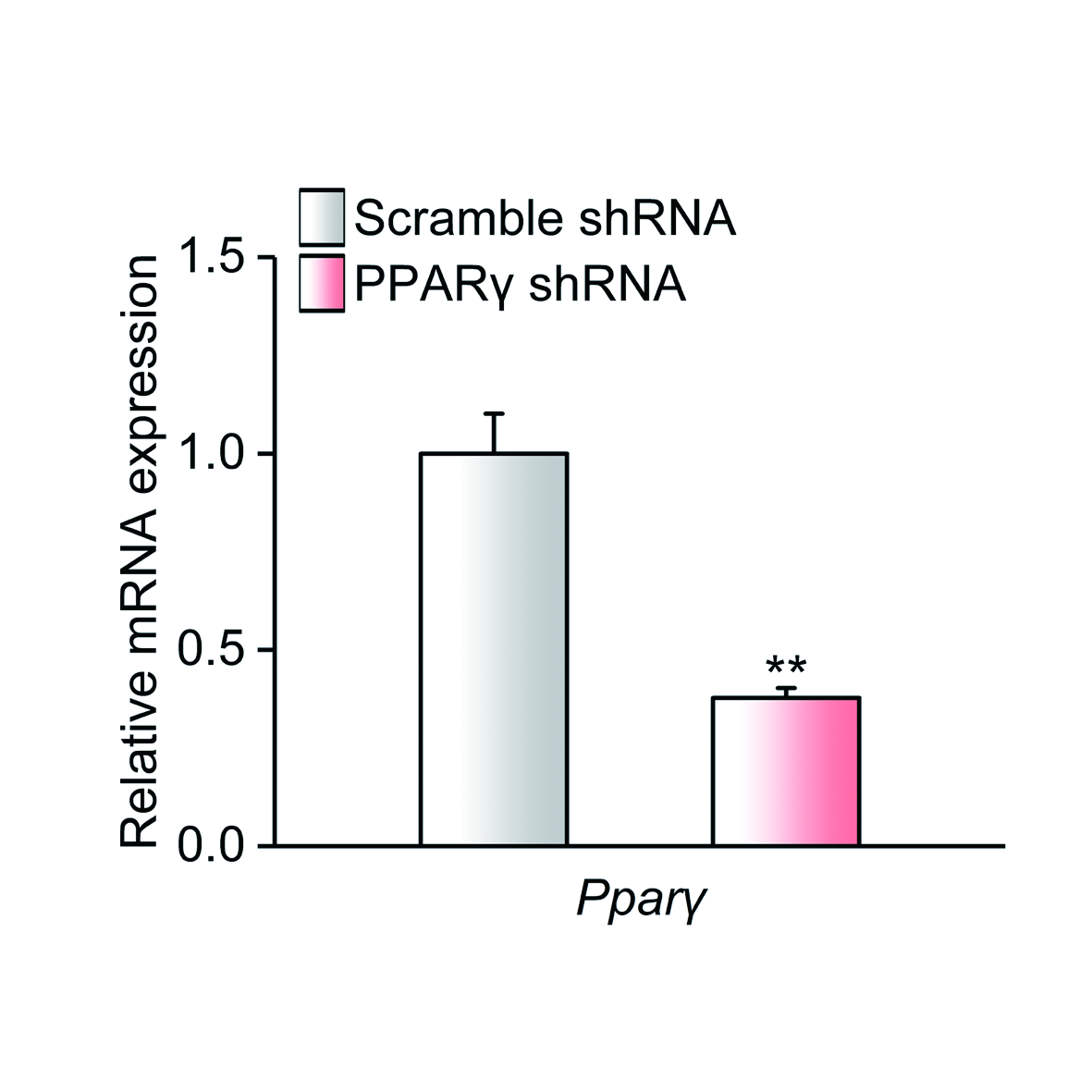

Supplement: Supplementary file 9 — Supplementary Figure 7 [file 41419_2019_1644_MOESM9_ESM.tif]

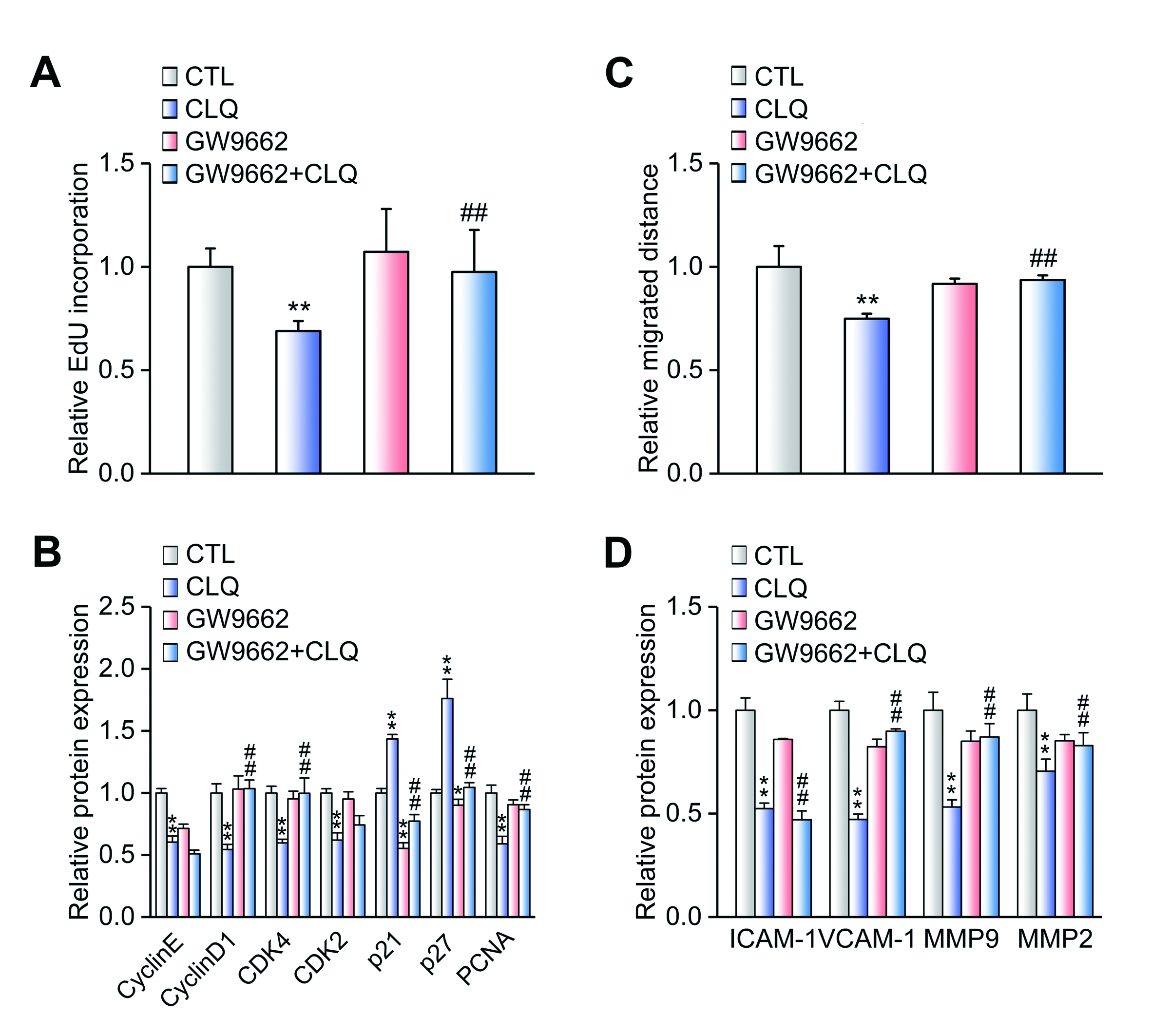

Supplement: Supplementary file 10 — Supplementary Figure 8 [file 41419_2019_1644_MOESM10_ESM.tif]

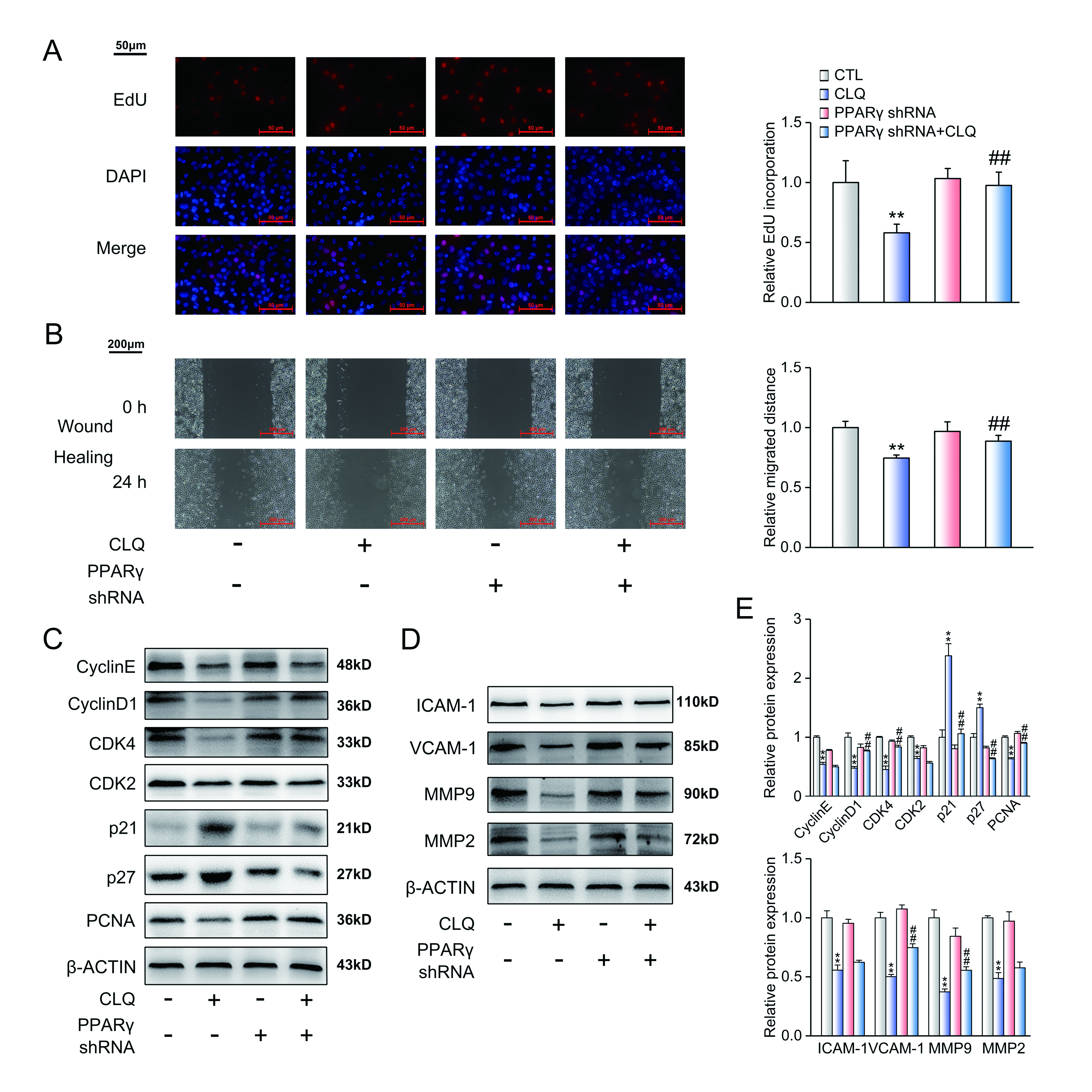

Supplement: Supplementary file 11 — Supplementary Figure 9 [file 41419_2019_1644_MOESM11_ESM.tif]

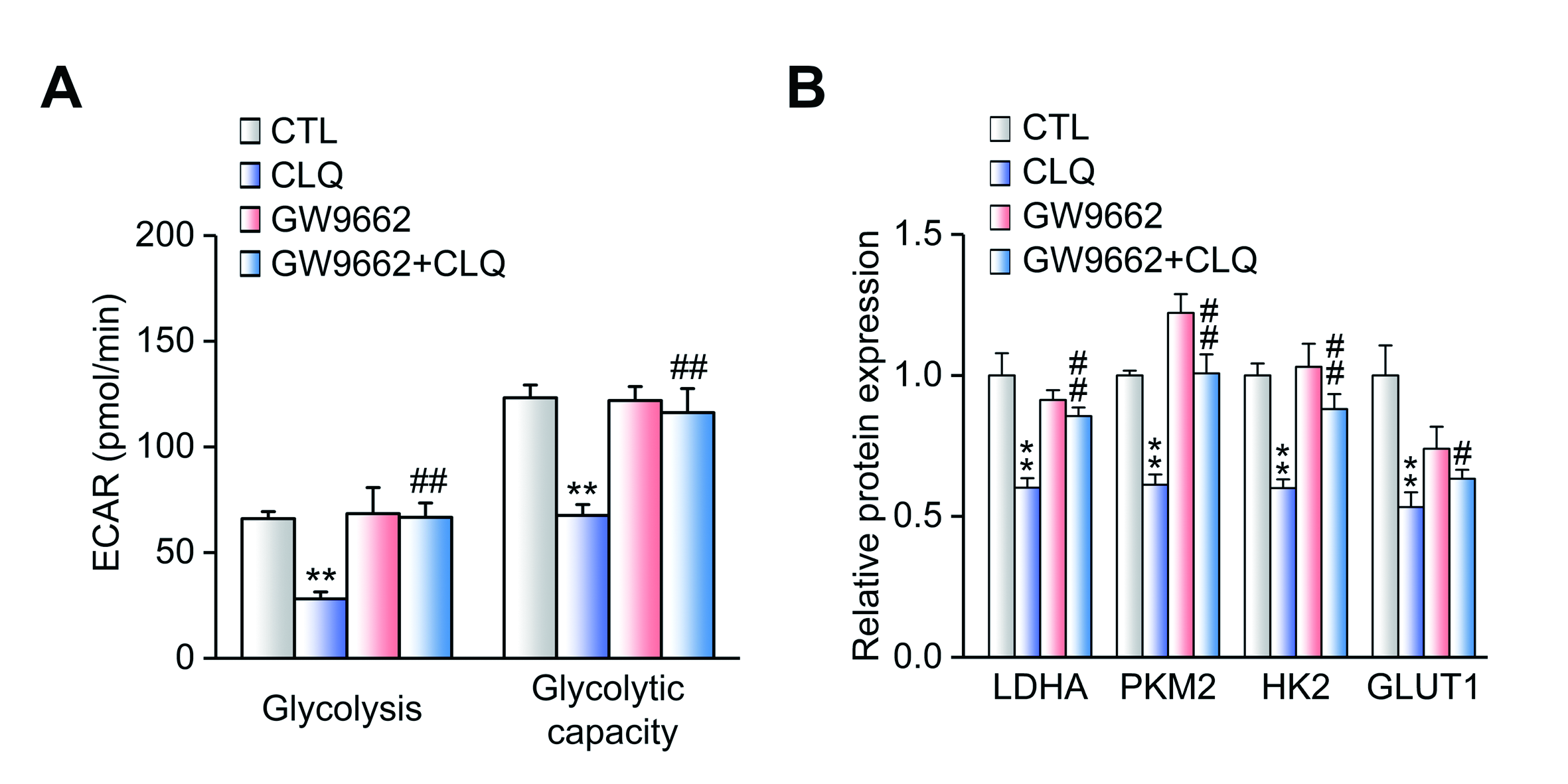

Supplement: Supplementary file 12 — Supplementary Figure 10 [file 41419_2019_1644_MOESM12_ESM.tif]
